# Characterisation of the Transcriptomes of Genetically Diverse *Listeria monocytogenes* Exposed to Hyperosmotic and Low Temperature Conditions Reveal Global Stress-Adaptation Mechanisms

**DOI:** 10.1371/journal.pone.0073603

**Published:** 2013-09-04

**Authors:** Juliana Durack, Tom Ross, John P. Bowman

**Affiliations:** 1 Department of Molecular and Cell Biology, University of California, Berkeley, California, United States of America; 2 Food Safety Centre, Tasmanian Institute of Agriculture, University of Tasmania, Hobart, Tasmania, Australia; University of Illinois at Chicago College of Medicine, United States of America

## Abstract

The ability of *Listeria monocytogenes* to adapt to various food and food- processing environments has been attributed to its robustness, persistence and prevalence in the food supply chain. To improve the present understanding of molecular mechanisms involved in hyperosmotic and low-temperature stress adaptation of *L. monocytogenes*, we undertook transcriptomics analysis on three strains adapted to sub-lethal levels of these stress stimuli and assessed functional gene response. Adaptation to hyperosmotic and cold-temperature stress has revealed many parallels in terms of gene expression profiles in strains possessing different levels of stress tolerance. Gene sets associated with ribosomes and translation, transcription, cell division as well as fatty acid biosynthesis and peptide transport showed activation in cells adapted to either cold or hyperosmotic stress. Repression of genes associated with carbohydrate metabolism and transport as well as flagella was evident in stressed cells, likely linked to activation of CodY regulon and consequential cellular energy conservation.

## Introduction

The opportunistic food-borne pathogen *Listeria monocytogenes* is equally adapted to life in the soil and life inside eukaryotic host cells. During its saprophytic life this bacterium can acquire tolerance to a vast array of physical and physiochemical stresses necessary to persist in the environment. Such stresses include elevated osmolarity (as high as 14%) [1–3] and cold temperature (a slow as -0.1°C) [4]. Ironically, similar stress hurdles are often used in food production to limit microbial proliferation and extend food shelf-life. Importantly, such exposures to hostile environments have been shown to encourage the development of cross-protection against stresses other than those used to limit propagation [5–9]. This creates a potential dilemma for proliferation control of this organism to prevent high enough numbers capable of causing life-threatening infection in immunocompromised individuals who consume contaminated food.

In depth comprehension of the molecular mechanisms behind stress adaptation in *L. monocytogenes* is therefore vital for optimizing control of its proliferation in food and consequently reducing the incidence of food-borne listeriosis.

Tolerance to physical stresses has been a topic of numerous investigations, both from a physiological basis and more recently from a genomic perspective [10–13]. Most publications focus on a single strain of *L. monocytogenes* [10,12–15]. Although this approach provides a necessary insight into molecular mechanisms of stress adaptation, it does not provide the full picture of the physiology of stress response adaptation in the organism. In this study, broad stress adaptation response in strains of *L. monocytogenes* possessing different tolerances to hyperosmotic and cold stress conditions was elucidated by cultivation and gene expression-based approaches. We show that *L. monocytogenes* tolerance and subsequent response to environmental stress is highly strain specific, though the physiological adjustments made are broadly similar between strains as well as between stress conditions.

## Materials

### Bacterial strains

A total of 148 strains of *L. monocytogenes* were used in this study. The collection contained 26 isolates of clinical origin, 30 isolates from food and food processing environments and 85 isolates of animal origin. The strains were acquired from the Medical Microbiological Diagnostic Unit, University of Melbourne, Victoria, Australia; from the Campden and Chorleywood Research Association, Chipping Campden, Gloucestershire, United Kingdom and Department of Medical Microbiology, Royal Hobart Hospital, Tasmania, Australia. Isolates from smoked salmon fillet samples and factory environment isolates were obtained from University of Tasmania Food Safety Centre, and the animal-derived isolates were collected by the former Department of Primary Industries in Tasmania from 1962 to 1990. The reference strains utilised included EGD (ATCC BAA-679), ATCC 19111, ATCC 19112, ATCC 19113, ATCC 19114, ATCC 19115, ATCC 7644, LO28, 10403S and ScottA. Strains were stored at -80°C in brain heart infusion broth (BHIB, Oxoid CM0225B) containing 15% (v/v) sterile glycerol.

### Salt stress tolerance experiments

*L. monocytogenes* strains were routinely cultivated on Brain Heart Infusion Agar (BHIA, Oxoid CM0375B) at 25°C for 24 h. One colony from each strain was inoculated in BHIB in duplicate and incubated for 24 h at 25°C. Ten microlitres of this culture was then aseptically transferred into L-shaped spectrophotometer tubes (‘L-tubes’) containing 10ml BHIB supplemented with 12.5% w/v NaCl (2.14M NaCl). Cultures were placed in a shaking incubator (model TN3; Advantec, Toyi Roshi international) in a constant temperature room (25±1°C) and transmittance was monitored every 1 h at 600nm (Spectronic 20, Milton Roy Co) until stationary phase was reached. Data obtained was analysed using a logistic growth model [16] LISREL (Scientific Software International, SSI) to solve for μmax (h^-1^) and maximum cell density and determine root mean square deviation.

### Cold (4°C) growth experiments

*L. monocytogenes* strains were routinely cultivated on BHIA at 25°C for 24 h. One colony from each strain was inoculated into BHIB and incubated for 24 h at 25°C. The cultures were diluted 1: 10^4^ in a fresh BHIB in quadruplicate. Microtiter trays (96 well, Eppendorf, Soth Pacific Pty. Ltd) were then inoculated with 200 µL of this suspension. Trays were sealed with sterile PCR adhesive film (Abgene) and incubated at 4±1°C, with temperature being monitored with a data logger. Change in absorbance was monitored using a BioRad Benchmark microplate reader at 540 nm until stationary phase was reached. Growth curves, following a log conversion of the OD data, were generated using the curve-fitting [17] DMFit software package (Institute of Food Research, IFR, UK).

### Population analysis

Experimental generation times (GT) for both stresses were re-sampled randomly with replacement using a bootstrap technique [18]. Bootstrapped population analysis was evaluated by constructing histograms based on percentage frequency of a mean GT within the 5000 bootstrap replicates based on the origin of isolate.

### cDNA Microarray-related treatments

For microarray experiments the strains investigated included those possessing a range of NaCl tolerances; strains ScottA (serotype 4b a clinical reference strain, possessing comparatively high NaCl tolerance but low tolerance for 4°C), ATCC19115 (serotype 4b a clinical reference strain, possessing moderate NaCl tolerance with a relatively high tolerance for 4°C) and 70-1700 (serotype 4e of animal origin isolated from ovine listeriosis case with relatively low NaCl and low temperature tolerance).

The three strains were grown in 50 mL BHIB under three different sets of conditions: unstressed, salt stressed and cold temperature stressed (4±1°C). All strains were grown to late-exponential-early stationary growth phase (*A*_*540*_ = ~0.6) in a Ratek shacking water bath (100 oscillations min^-1^) in 200-mL sidearm flasks that allowed for regular monitoring of cell suspension turbidity. Unstressed, control strains were grown at 25°C; cold stressed cells were grown at 4°C; salt stressed cells were grown at 25°C in BHIB supplemented with 8% w/v NaCl for strain 70-1700, 10% w/v for ATCC 19115 and 12% w/v NaCl for ScottA. To stabilise the cellular RNA content, the cultures were treated with RNA Protect reagent (Qiagen) according to manufacturer’s protocol. All experiments were replicated on separate days resulting in two biologically replicated samples for each strain at three different conditions.

### RNA extraction

Cells were thawed on ice and underwent a 6 h enzymatic treatment in 10 mM Tris-1 mM EDTA buffer (pH 8.1) containing 20 mg/ml lysozyme (Sigma-Aldrich, L7651) and 10 mg/ml proteinase K (Sigma-Aldrich, P2308) at 25°C. Cells were fully lysed by bead beating using 0.1 mm zirconium-silica sand in 4 ml of RNeasy Midi RNA Extraction kit (Qiagen) lysis buffer that was supplemented with 0.1% β-mercaptoethanol (Sigma-Aldrich). RNA was then extracted and purified using RNeasy Midi RNA Extraction kit (Qiagen). RNA quality and quantity was assessed by running the RNA samples on a formaldehyde (FA) gel containing 1.2% w/v agarose (5-7 V/cm in 1xFA gel running buffer).

### Microarray analysis

RNA samples were hybridized to a microarray slide containing *L. monocytogenes* AROS version 1 oligonucleotide set (Eurofins MWG Operon, Huntsville, Al, USA), representing all predicted protein coding genes and pseudogenes of the complete, published genome of *L. monocytogenes* EGD-e (GenBank accession number AL591824). Oligonucleotides were arrayed onto glass slides using quill pens at the Australian Genomic Research Facility Ltd. (Walter & Eliza Hall Institute of Medical Research, Parkville, Victoria, Australia) with each spot possessing on average a 12 µm diameter. Approximately 5-20 µg of total RNA was converted to cDNA and postlabelled using Cy5 (red) and Cy3 (green) fluorescent dyes and a SuperScript III indirect cDNA labeling system (Invitogen) in a series of steps previously described [19]. Slides were scanned using a GenePix 4000B scanner (Axon Instruments). Downstream processing used the GenePix-Pro 5.2 software package to generate GPX filed from TIFF array images. Global normalization of raw data followed the method previously described [19]. Oligonucleotides that showed negligible or no hybridization when compared with background hybridization were excluded from analyses. In addition genes only known to be found in the majority of *L. monocytogenes* genomes were considered in the analysis (if present in sequenced strains EGD-e, F2635, HCC23 and Clip 81459) if the given 70-bp oligonucleotide sequence had a similarity level of at least 92%.

### Gene expression trend analysis

Gene designation, predicted functions and categorization of coded proteins into defined sets from the *L. monocytogenes* EGD-e genome was based on information obtained from published literature, Kyoto Encyclopaedia of Genes and Genomes (http://www.genome.ad.jp/kegg/) and ListiList (http://genolist.pasteur.fr/ListiList/). A *T*-test based procedure previously described [19–22] was utilised to score the changes in expression of predefined sets of genes. The significance of the *T*-value score was established by using the associated two-tailed *P*-value determined with the TDIST function in Microsoft Excel using the number of genes minus 2 for degrees of freedom. In addition to functional categories, the *T*-value scores for gene sets equivalent to the regulons that comprise genes under the influence of transcriptional regulators SigB [13,23], CtsR [24], HrcA [25], PrfA [26] and CodY [27] were determined.

### Microarray data accession number

Microarray hybridization-derived information included in this report has been deposited under Gene Expression Omnibus (GEO) database accession number GSE46612.

### Motility assessment

Effect of salt and temperature on swarming motility was assessed using BHIA plates containing 0.3% w/v bacteriological agar and 0.05g/L filter sterilised 2,3,5-triphenyltetrazolium chloride (TTC). Various amounts of NaCl were added to BHIA to achieve salinity levels ranging from 3 to 8% NaCl (w/v). Plates were inoculated with a single colony (24 h growth on BHIA at 25°C) of one strain, with a maximum of four inoculations per plate and incubated at 25°C for 48 h or 4°C for 14 days. Diameter of zones of motility, observed as red swarming halos were measured in millimetres at the widest point with 1mm being an absolute minimum (size of the original stab), indicating zero swarming motility.

## Results and Discussion

Strain specific tolerance to various stresses has previously been noted in *Listeria monocytogenes* [3,28–31], where certain strains appear to be more or less adapted to specific stress. This was also evident in our investigation of a large assortment of isolates of diverse origin, where relatively high osmotic stress (2.14M or 12.5% w/v NaCl) and low temperature of 4°C were selected as a proxy for assessing low water activity and cold temperature tolerance of this organism.

### Evaluation of growth parameters

The selected high salt concentration of 12.5% (equivalent to a_w_ of 0.92 at 25°C) was sufficient to identify, and inhibit growth of salt sensitive isolates, and to notably challenge the remaining 84 salt tolerant isolates. Generation time (GT) varied significantly within the salt tolerant isolates ranging from as fast as 5.4 h to as slow as 15.7 h (Table S1). To evaluate distribution of stress tolerance we conducted population analysis based on origin of the isolates from our strain collection.

**Figure 1:**
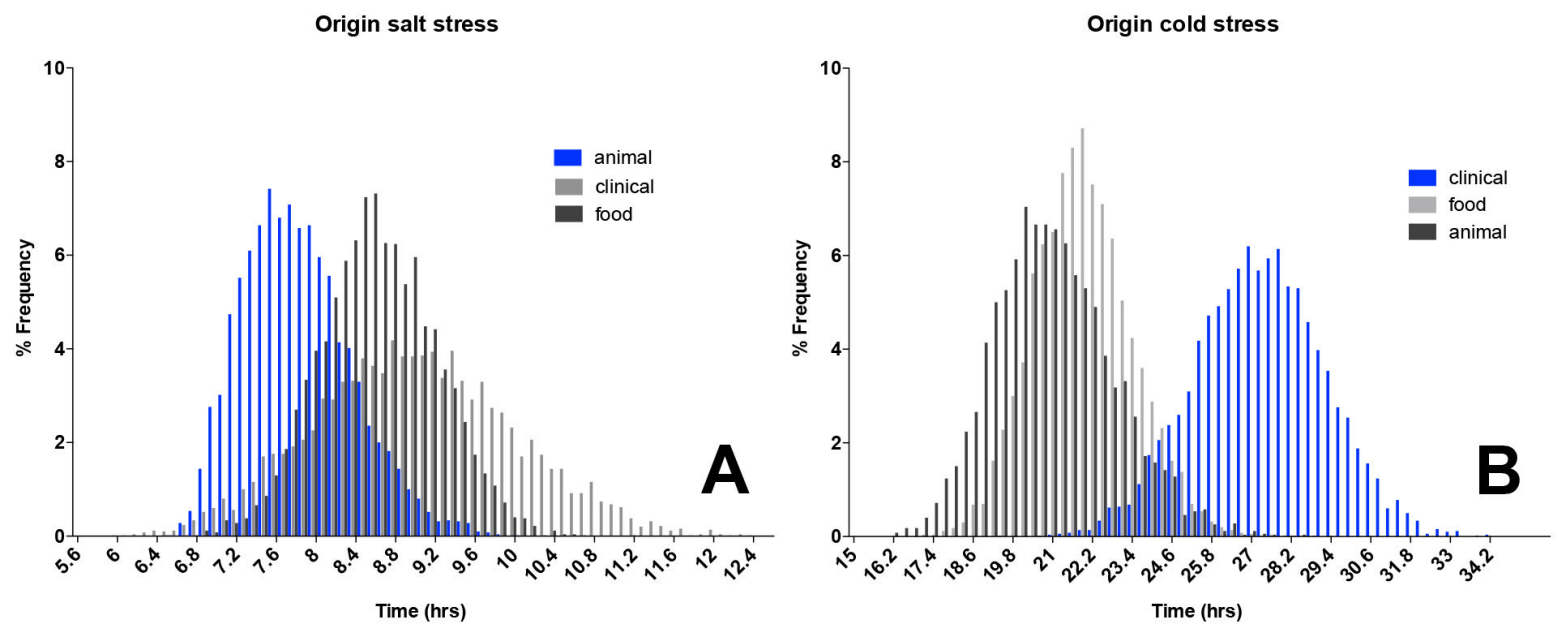
Histogram showing distribution of a mean generation time within bootstrap replicates based on the origin of isolate of *Listeria monocytogenes* strains exposed to either a.) 12.5% NaCl or b.) cold 4°C temperature in BHIB.

The majority of isolates able to proliferate at the selected high salt concentration originated from animal sources, with an average generation time of 7.7±0.56 h, suggesting higher tolerance for hyperosmotic stress than isolates of food or clinical origin (Figure 1a). Animal isolates of *L. monocytogenes* presumably reach the affected animal from natural environment, water or feed. The saprophytic existence of *L. monocytogenes* in natural environments may mean that strains isolated from animals were already preselected for stress tolerance due to variable and often adverse environmental conditions.

Extensive variation in GTs was also observed among tested isolates at 4°C, ranging from 9.9 h to 47.1 h (Table S1). These observations of a wide GT distribution are in agreement with other studies [30,32] emphasising variability between strains in response to environmental insult.

Strains of clinical origin exhibited significantly slower average GT of 27.2±2.0 h at 4°C than either animal or food isolates (Figure 1b). This is the first indication of a lower cold tolerance in clinical strains of *L. monocytogenes* potentially due to a large number of isolates tested in this study. Animal and food isolates in comparison produced on average faster GTs at 4°C 20.7±6.7 h and 21.7±5.0 h respectively; suggesting inherent adaptation to low temperature. This is likely due to more frequent exposure of these strains to cold stress. An ability to rapidly multiply in a cold environment could be of benefit to *L. monocytogenes* competing for nutrients with other microorganisms less adapted to proliferation at low temperature either in the environment or in a food matrix.

### Strain specific gene expression caused by adaptation to stress

To evaluate mechanisms responsible for the observed heterogeneity in adaptation to stress, strains possessing different levels of tolerance to hyperosmotic and cold stress were selected for transcriptomic analysis. Strains were exposed to sub-lethal levels of salt individually established for each strain, to elicit comparable levels of assault in order to provide a more rigorous analysis of responses to stress. To broaden our current understanding of cross-protection the same strains were evaluated for adaptation to cold temperature. Adaptation to stress rather than shock response was evaluated. Adaptation to stressful conditions renders bacteria which have re-established the disrupted homeostasis with their external environment (as opposed to shock stress), resuming cellular functions such as DNA and protein synthesis, cell growth and cellular division. In terms of food safety, bacteria in a stress-adapted physiological state presumably cause the most threat, as these are able to multiply in the food matrix reaching high enough numbers to cause infection upon consumption by susceptible individuals.

Analysis of gene expression in cells adapted to cold and hyperosmotic stress revealed significant strain specificity in terms of individual gene expression profiles though general patterns of expression were largely similar.

Strain ATCC19115 revealed 150 significantly up- and 146 down-regulated genes in cells adapted to both hyperosmotic and independently to cold stress (Tables S2 and S3 respectively), ScottA showed up-regulation of 77 genes whilst 106 genes were significantly down-regulated (Tables S4 and S5 respectively), whereas 82 up- and 56 down-regulated genes were matching in response to both stresses in strain 70-1700 (Tables S6 and S7 respectively). Among these homologously expressed genes only 22 were found to be significantly up-regulated (Table 1) and only six were down-regulated (Table 2) in all three strains adapted to hyperosmotic stress and cold temperature.

**Table 1:** Log ratios of significantly up-regulated genes in *L. monocytogenes* strains independently adapted to sub-lethal levels of NaCL and 4°C.

Gene	Gene homolog	ATCC19115		ScottA		70-1700		Predicted or known function
		NaCl	4°C	NaCl	4°C	NaCl	4°C	
*lmo0198*	*gcaD*	2.10^***^	2.35^***^	1.20^***^	1.12^**^	1.34^***^	1.15^***^	glucosamine-1-phosphate N-acetyltransferase / UDP-N-acetylglucosamine pyrophosphorylase
*lmo0227*		3.21^***^	1.88^***^	2.68^***^	1.31^***^	1.32^**^	1.64^***^	putative tRNA-dihydrouridine
*lmo0248*	*rplK*	3.36^***^	2.25^***^	1.49^***^	2.54^**^	1.79^***^	3.16^***^	ribosomal protein L11
*lmo0249*	*rplA*	3.37^***^	1.71^**^	1.90^***^	2.01^**^	1.08^**^	2.97^***^	ribosomal protein L1
*lmo1267*	*Tig*	1.82^***^	2.21^**^	1.10^**^	1.85^*^	1.59^***^	1.01^*^	trigger factor (prolyl isomerase)
*lmo1431*		2.67^***^	3.06^***^	2.02^***^	2.10^***^	1.37^***^	1.97^***^	ABC transporter, ATP-binding protein
*lmo1596*	*rpsD*	3.21^***^	1.32^***^	1.03^***^	1.73^*^	1.48^***^	1.99^***^	ribosomal protein S4
*lmo1707*		1.95^***^	1.56^**^	1.10^***^	1.81^***^	1.08^***^	1.26^***^	unknown protein
*lmo1784*	*rpmI*	2.39^***^	1.32^***^	1.80^***^	1.66^**^	1.38^***^	2.32^***^	ribosomal protein L35
*lmo1816*	*rpmB*	3.27^***^	4.34^***^	3.01^***^	3.58^**^	1.84^***^	4.11^***^	ribosomal protein L28
*lmo2048*		2.70^***^	1.15^**^	2.59^***^	1.75^***^	1.64^***^	2.09^***^	similar to uncharacterized conserved proteins
*lmo2056*		1.43^**^	1.47^***^	1.66^***^	1.48^***^	1.44^***^	1.51^***^	similar to uncharacterized conserved proteins
*lmo2192*	*oppE*	2.11^***^	1.18*	2.26^***^	1.15^*^	1.63^***^	1.85^***^	similar to oligopeptide ABC transporter, ATP binding protein
*lmo2194*	*oppC*	1.44^**^	1.82^*^	1.46^***^	1.31^*^	1.43^***^	1.74^***^	similar to oligopeptide ABC transporter, permease protein
*lmo2195*	*oppB*	1.62^**^	1.63^***^	1.38^***^	1.71^**^	1.17^***^	1.72^***^	similar to oligopeptide ABC transporter, permease protein
*lmo2223*		2.41^***^	2.28^**^	2.74^**^	1.89^*^	1.37^***^	1.10^***^	similar to uncharacterized conserved proteins
*lmo2376*	*ppiB*	1.92^**^	1.96^***^	1.27^***^	1.25^**^	1.39^***^	1.20^***^	similar to peptidyl-prolyl cis-trans isomerase
*lmo2428*		2.02^***^	1.64^***^	1.08^***^	2.12^***^	1.24^***^	2.47^***^	similar to FtsK/RodA/SpoIIIE and related proteins
*lmo2479*		1.44^*^	1.13^*^	1.14^***^	1.27^***^	1.47^***^	1.31^***^	similar to uncharacterized conserved proteins
*lmo2522*		2.11^**^	6.55^***^	3.90^***^	4.40^***^	3.52^***^	4.30^***^	similar to uncharacterized conserved proteins
*lmo2548*	*rpmE*	2.59^***^	2.49^***^	1.95^***^	3.32^**^	1.91^***^	3.69^***^	ribosomal protein L31
*lmo2555*		1.68^***^	2.55^***^	1.15^***^	1.68^**^	1.13^***^	1.09^***^	similar to glycosyltransferases

Genes were considered significantly up-regulated with log ratio >1 which is equivocal of twofold up-regulation. * indicates statistical significance of Log ratio for each gene, where *P<0.05, **P<0.01, ***P<0.001. Gene nomenclature used as per *L. monocytogenes* EGD-e genome. Gene homologs and predicted functions were obtained collectively from variety of sources including circulating literature and web based databases.

**Table 2:** Log ratios of significantly down-regulated genes in *L. monocytogenes* strains independently adapted to sub-lethal levels of NaCl and 4°C.

Gene	Gene homolog	ATCC19115		ScottA		70-1700		Predicted or known function
		NaCl	4°C	NaCl	4°C	NaCl	4°C	
*lmo0265*		-3.11^**^	-2.03^**^	-2.28^***^	-3.49^**^	-1.91^***^	-3.34^***^	putative succinyl-diaminopimelate desuccinylase
*lmo0445*		-2.67^**^	-1.22^*^	-1.29^***^	-1.10^**^	-1.44^***^	-1.09^***^	similar to transcriptional regulators/antiterminators
*lmo0497*		-1.47^**^	-1.17^**^	-1.71^*^	-1.54^***^	-1.57^***^	-1.38^***^	similar to glycosyltransferases
*lmo2363*	gadB	-3.97^***^	-4.26^***^	-5.11^***^	-4.69^***^	-1.05^*^	-2.63^**^	glutamate decarboxylase
*lmo2684*		-9.46^***^	-4.14^***^	-3.09^**^	-1.11^**^	-1.58^**^	-2.85^***^	similar to PTS system, cellobiose-specific IIC component
*lmo2685*		-8.68^***^	-6.00^***^	-2.01^***^	-1.42^**^	-1.23^***^	-2.86^***^	similar to PTS system, cellobiose-specific IIA component

Genes were considered significantly down-regulated with log ratio <-1 which is equivocal of twofold down-regulation. * indicates statistical significance of Log ratio for each gene, where *P<0.05, **P<0.01, ***P<0.001. Gene nomenclature used as per *L. monocytogenes* EGD-e genome. Gene homologs and predicted functions were obtained collectively from variety of sources including circulating literature and web based databases.

In order to gain insight into physiological adjustments to the set stress conditions, we grouped genes on the bases of a functional role assigned through comparisons to bioinformatic databases and direct experimental data. Analysis of gene expression utilizing a combined ontological- and statistical-based approach [22] allowed identification of trends between functionally linked genes, providing an overview of a global stress response mechanisms.

### Adaptation to hyperosmotic and cold stress induced translation-apparatus related genes

**Figure 2:**
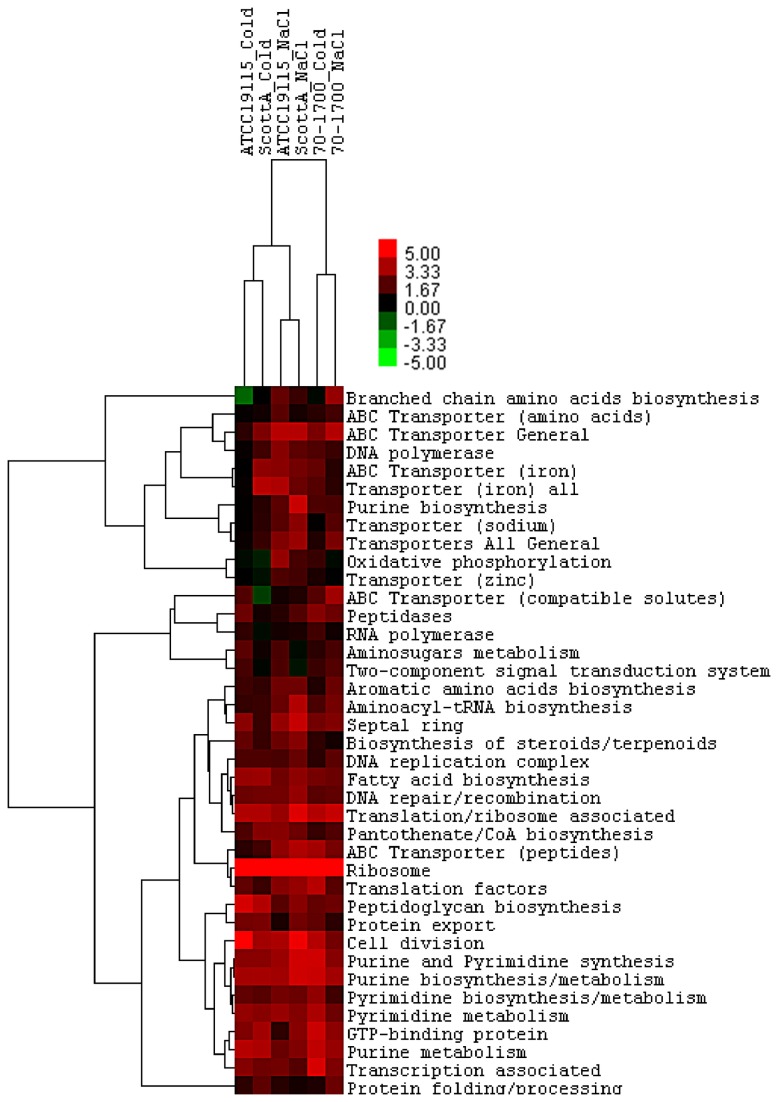
Heat map showing statistical trends for up-regulated functionally defined sets of genes in strains of *L. monocytogenes* exposed to either sub-lethal level of NaCl or cold stresses. T-test based procedure was used to score the changes in expression of each functional gene set, statistically significant T-values (as determined by a two-tailed P-value) are expressed from -5 (shown in green) to 5 (shown in red). Gene sets were considered significantly up-regulated with T-value ≥ 1 and significantly down-regulated with T-value ≤ -1.

Adaptation to hyperosmotic and cold stress had a stimulating effect on translation-apparatus related genes. Genes associated with ribosomes were among the most strongly activated gene sets in response to both stresses with average *T*-value scores of 10.46 (±0.58) for salt adapted strains and 10.65 (±3.25) for cold adapted strains (Figure 2). Among a large array of up-regulated ribosomal genes eight showed consistently strong (>two fold) homologous up-regulation in all three strains following independent adaptation to both stresses (Table 1) these were *rplK* (*lmo0248*), *rplA* (*lmo0249*), *rpsD* (*lmo1596*), *rpmI* (*lmo1784*), *rpmB* (*lmo1816*) and *rpmE* (*lmo2548*). Although different physical stresses, both high Na^+^ concentration and low temperature have a damaging effect on ribosome function. While high ionic concentration is thought to cause the ribosome subunits to become displaced from mRNA strands [33], low temperature alters the structural integrity of ribosomal subunit [34], both resulting in stalling of translation. Stress response adaptation of *L. monocytogenes* to both stress factors appears to involve activation of ribosomal gene transcription. Gene sets encoding proteins involved in assisting protein folding and translation overall were evidently affected by both stresses with significant activation of gene sets associated with these cellular processes. This could be due to interference in protein folding, induced independently by high levels of sodium cations and low temperature. Two genes *ppiB* (*lmo2376*) and *tig* (*lmo1267*) were universally up-regulated in cells adapted to either of the two stress factors (Table 1). Both cyclophilin (PpiB) [35] and Tig (trigger factor) [36,37] catalyse cis-trans isomerization of peptide bonds and are important for correct folding of nascent proteins [38]. Mutants of *L. monocytogenes* lacking Tig showed susceptibility to ethanol stress [39] and activation of both trigger factor and cyclophilin are essential for growth under starvation conditions [38] and during cold-shock response [35] in *Bacillus subtilis* as well as the survival of a soil bacterium *Sinorhizobium meliloti* under hyperosmotic conditions induced by NaCl [40,41].

### Adaptation to hyperosmotic and cold stress induced transcription associated genes

The structural integrity of DNA molecules is also sensitive to changes in the physiological environment, which may explain the enhancement of gene transcription associated with DNA repair, recombination and other proteins involved in DNA stabilization in *L. monocytogenes* exposed to environmental stress (Figure 2). High salt concentration causes dehydration of the DNA molecule in which water molecules are displaced by sodium cations, which in turn disturbs electrostatic interactions of DNA [42], whilst low ambient temperature directly alters DNA structure by increasing negative supercoiling [43]. *L. monocytogenes* appears to respond to changes to the DNA molecule structure by up-regulating genes that encode support proteins responsible for maintaining the integrity of DNA. Interestingly, both stresses induced activation of different individual genes within this functional group of genes. Whilst activation of *ssb* (*lmo0045*), encoding single-stranded DNA-binding protein, and *hsdM* (*lmo1582*), encoding a DNA methylase, was evident in all strains during adaptation to hyperosmotic stress, growth at cold temperature activated *topA* (*lmo1275*)*, parE* (*lmo1286*), *nusG* (*lmo0246*), *holB* (*lmo0162*) and *cspL* (*lmo1364*) in all strains of *L. monocytogenes* tested (Tables S2, S4 and S6). Accumulation of a single stranded-DNA-binding protein, Ssb has been shown to occur in *E. coli* cells after an increase of the extracellular NaCl concentration and is believed to play a role in protection and repair of chromosomal DNA during cellular stress [44]. An increase in negative supercoiling of DNA molecule induced by cold temperature, would hinder the transcription machinery in bacterial cells and needs to be overcome to allow for undisturbed cell metabolism during cold stress. At least in *E. coli* the superhelical tension of DNA molecule encountered at low-temperature is mainly regulated through the two opposing topoisomerase activities [43]. Up-regulation of topoisomerase encoding genes, *parE* and *topA* observed, suggests a potential involvement of these in maintaining superhelical tension in this organism under cold stress. Significant activation of transcription antitermination factor NusG is known to be induced by cold shock through transcription anti-termination mediated by cold shock protein A (CspA) and other cold shock proteins in *E. coli* [45]. This together with activation of DNA polymerase subunit encoded by *holB*, suggests enhanced transcription requirement by cold-adapted cells, perhaps to maintain transcription levels sufficient for undisrupted cell functions. Activation of CspL in *L. monocytogenes* strain 10403S has been observed in both log- and stationary growth phase at 4°C [46] and in cold adapted strain LO28 at 10°C [47]. Involvement of cold stress proteins in adaptation to cold stress has not been fully resolved however it is accepted that these proteins assist cell adaptation to low temperature through RNA-chaperone activities [34,48,49]. This presumably aids transcription and translation functions that are hindered under low-temperature stress.

### Adaptation to hyperosmotic and cold stress induced changes to cell envelope and membrane transport

Adaptation to hyperosmotic and cold environments was evidently accompanied by a change in the cellular envelope. Gene sets encoding proteins associated with peptidoglycan biosynthesis showed significant activation in *L. monocytogenes* adapted to either hyperosmotic or cold stress (Figure 2). This was accompanied by transcriptional up-regulation of genes linked to fatty acid biosynthesis, most likely associated with cell membrane structure modulation occurring in response to both stress factors.

It was interesting to note that one gene, *lmo0198*, homologous to *gcaD* in *B. subtilis* [50] and *glmU* in *E. coli* [51], which encodes *N*-acetylglucosamine 1-phosphate uridyltransferase responsible for synthesis of peptidoglycan precursor UDP-N-acetylglucosamine, was up-regulated in all strains following adaptation to both stresses. Involvement of this gene transcript in adaptation to cold and salt environments in *L. monocytogenes* has been overlooked in previous studies; however strong transcriptional up-regulation of this gene observed throughout this study suggests an important role in stress adaptation of *L. monocytogenes* in hyperosmotic and cold temperature environments.

Another highly expressed gene was *lmo2522* it showed a relatively strong up-regulation (up to 93-fold) in strains adapted to both cold and salt stimuli (Table 1). The encoded resuscitation promoting factor (Rpf) [52], homologous to YocH in *B. subtilis*, is a cell membrane binding protein with muralytic activity. Transcriptional up-regulation of this Rpf has been linked, to salt stress response in *B. subtilis* [53]; cold adaptation in *L. monocytogenes* strain 10403S [46], prolonged exposure to ethanol in starin LO28 [54] and more recently to antibiotic induced cell envelope stress [55]. A recently characterised gene, *lmo2555* also showed up-regulation, which encodes cytoplasmic glycosyltransferase, LafA (LTA anchor formation protein A) involved in the synthesis of the glycolipid Gal(α1-2) Glc(α1-3)-diacylglycerol (Gal-Glc-DAG), the anchor which connects LTA to the bacterial membrane [56,57]. Deletion of *lmo2555* led to a complete absence of glycerolipids in *L. monocytogenes* strain 10403S [56].

Bacterial interaction with the environment adaptation to stressful challenges is controlled by the viscosity of the cell membrane incorporating fatty acid structures into the component phospholipids which in turn modulates permeability [58]. Both environmental stresses examined in this study induced modification of cell membrane composition in *L. monocytogenes* and stimulated significant change in transporter related gene transcription observed in adaptive response of this organism to both stresses.

Adaptation to both stresses appeared to activate transcription of genes associated with general transport across the cell membrane, in particular those associated with uptake of peptides (Figure 2). Up-take of peptides from the external environment as a means of hyperosmotic [59] and cold [60] stress acclimatisation has previously been described in *L. monocytogenes*. Indeed the *oppA* operon overall showed significant up-regulation in all strains adapted to either cold or hyperosmotic stresses, thus further emphasising the involvement of the encoded oligo-peptide transporter in aiding adaptation to environmental stress in this pathogen. The Opp systems of other bacteria are thought to be involved in nutrient uptake [60,61] and recycling the cell membrane peptides for synthesis of new peptidoglycan [62]. In addition, accumulation of oligopeptides from the growth medium, such as peptides containing glycine and proline derivatives, may aid stress tolerance in a way similar to that observed for compatible solutes [63]. Accumulation of compatible solutes as a mechanism of overcoming hyperosmotic or cold temperature stress in *L. monocytogenes* has been closely examined in numerous independent studies. It is well accepted that compatible solutes provide stability to enzymes and assist protein folding and other cellular processes vulnerable to environmental stress, prevent cold induced aggregation of proteins as well as maintaining cell volume by counteracting water efflux [64–66]. Genes encoding compatible solute transporters collectively showed up-regulation in *L. monocytogenes* cells adapted to cold and also osmotic stress (Figure 2), however strain specificity for compatible solute preference was significant and something that has not been previously addressed in stress response adaptation of this pathogen. One exception was significant up-regulation of *betL* (*lmo2092*) in all strains during adaptation to salt stress only, as no change in transcription levels were evident in cold adapted cells. The BetL transporter is the secondary betaine transporter driven by the membrane potential of the cell and the ionic strength of the growth medium [67,68]. BetL expression is independent of σ^B^ and it is known to be responsible for the majority of glycine betaine uptake in *L. monocytogenes* immediately following osmotic shock, and long-term protection for low levels of stress, however high levels of NaCl previously have been shown to suppress this transporter [68–70]. Here we show that transcription of *betL* is activated in strains adapted to high levels of NaCl, suggesting continuous uptake of the compatible solute glycine betaine via the BetL permease in these cells.

Interaction with the external environment is an important aspect of bacterial stress response and it appears that *L. monocytogenes* not only actively accumulates substances from the external environment but also actively exports proteins during adaptation to stressful environments. The protein secretion systems in *L. monocytogenes* are not completely understood [71], especially those involved in stress adaptation. It is highly likely that activation of protein secretion mechanisms, observed in both cold and salt adapted cells (Figure 2), is associated with translocation of membrane proteins responsible for maintaining the integrity of the cell envelope. Whether this up-regulation is linked to an increase in protein turnover associated with physical damage to the cell membrane, and consequently, an increased demand for newly synthesised proteins, or for translocation support proteins contributing to stabilising the secreted protein, remains to be determined.

### Adaptation to hyperosmotic and cold stress induced activation of genes involved in cell division

Bacterial growth relies on a complex assembly of cell division proteins, the correct alignment of which is crucial for generating viable daughter cells. It is not surprising that such a finely tuned cellular process is quite vulnerable to environmental stress that can, if not corrected, disrupt the balance of cell septation and, thus, compromise the overall process. Gene sets associated with cell division and septal ring formation showed up-regulation in *L. monocytogenes* cells adapted to cold and hyperosmotic environments (Figure 2).

Low temperature and high ionic strength induced by NaCl appeared to influence the function of the cell division machinery (Figure 2), possibly through compromising the correct folding and, consequently, the structure and stability of cell division proteins. Vulnerability of cell division proteins to low temperature and hyperosmotic environment has also been observed in other bacteria [72–75]. Transcriptional up-regulation of septal ring stability factor encoding genes *ftsX* and *ftsE* [76] observed in osmoadapted cells along with *minCD* encoding cell division inhibitors [77] evident in cold adapted cells, may function in preventing improper and premature assembly of the division machinery in stress adapted cells.

### Adaptation to hyperosmotic and cold stress induced suppression of genes involved in carbohydrate metabolism

**Figure 3:**
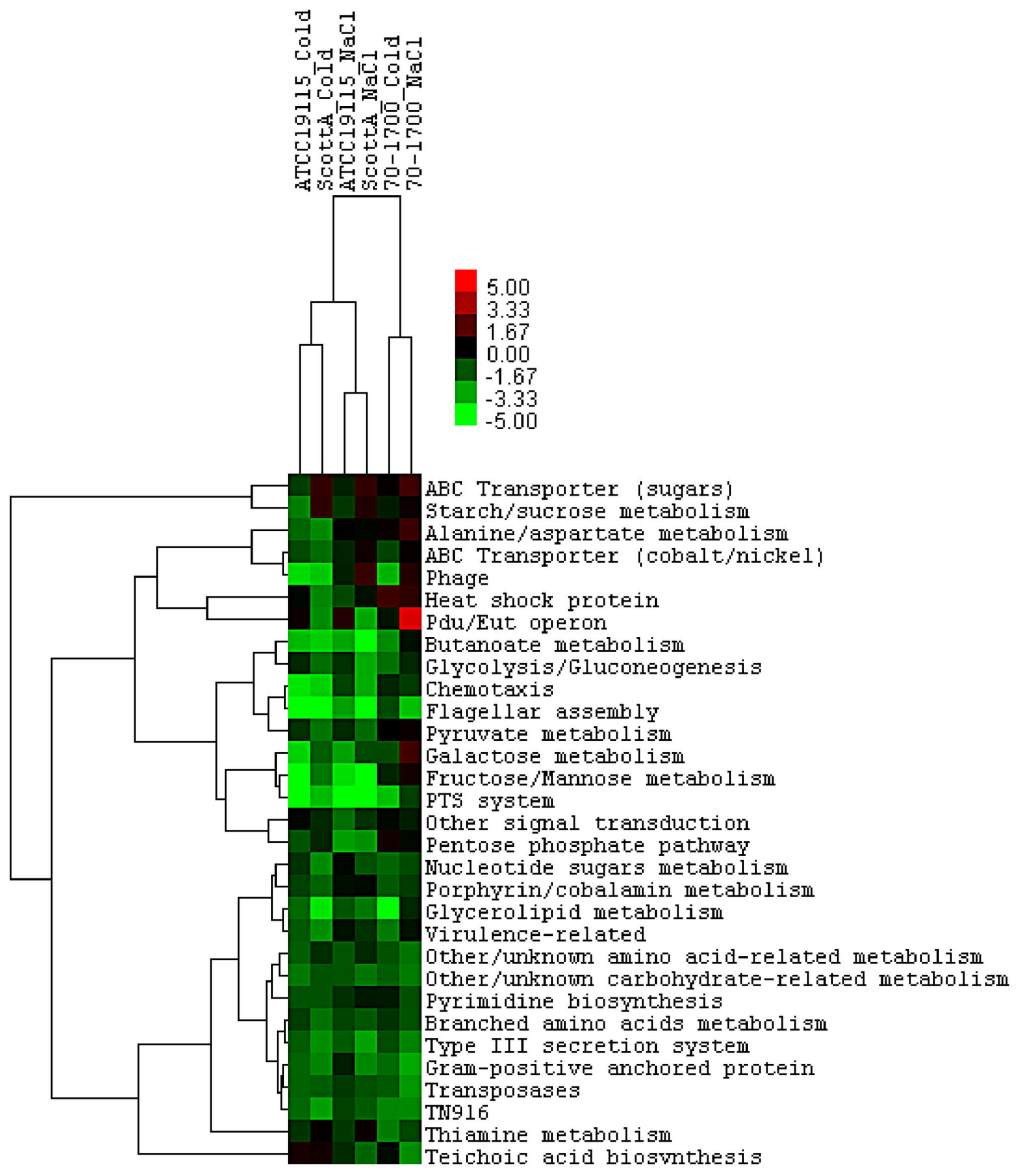
Heat map showing statistical trends for down-regulated functionally defined sets of genes in strains of *L. monocytogenes* exposed to either sub-lethal level of NaCl or cold stresses. T-test based procedure was used to score the changes in expression of each functional gene set, statistically significant T-values (as determined by a two-tailed P-value) are expressed from -5 (shown in green) to 5 (shown in red). Gene sets were considered significantly up-regulated with T-value ≥ 1 and significantly down-regulated with T-value ≤ -1.

Stress adaptation in *L. monocytogenes* appears to be accompanied by a decrease in overall metabolic turnover, characterised by the evident slowing of growth. This was reflected in overall down-regulation of gene sets associated with carbohydrate transport and utilization observed in cold and osmoadapted cells (Figure 3). Gene sets associated with phosphotransferase systems were strongly suppressed in *L. monocytogenes* cells adapted to cold and salt stresses. This corresponds to suppression of 3.2% of total *L. monocytogenes* genes, in turn restricting wastage of phosphodonor compounds on nonessential metabolic processes such as up-take of sugars, utilisation of which is suppressed due to overall metabolic slowdown.

### Adaptation to hyperosmotic and cold stress induced suppression of genes involved in chemotaxis and other external membrane proteins

Flagella motility is a highly advantageous but energetically demanding survival mechanism utilised by bacteria in extracellular environments [78]. Adaptation to cold and salt stress lead to suppression of gene sets associated with flagella assembly and chemotaxis (Figure 3).

Most published literature on flagella proteins and their transcriptional regulation address the involvement of flagella in *L. monocytogenes* virulence. There is limited information, however, regarding the effects of environmental stress such as cold-temperature and NaCl on motility particularly in nutrient rich environment such as brain heart infusion (BHI) medium. To validate the observed flagella structural and biosynthesis-associated gene expression we investigated the effect of various levels of NaCl and low temperature on phenotypic swarming motility response.

**Figure 4:**
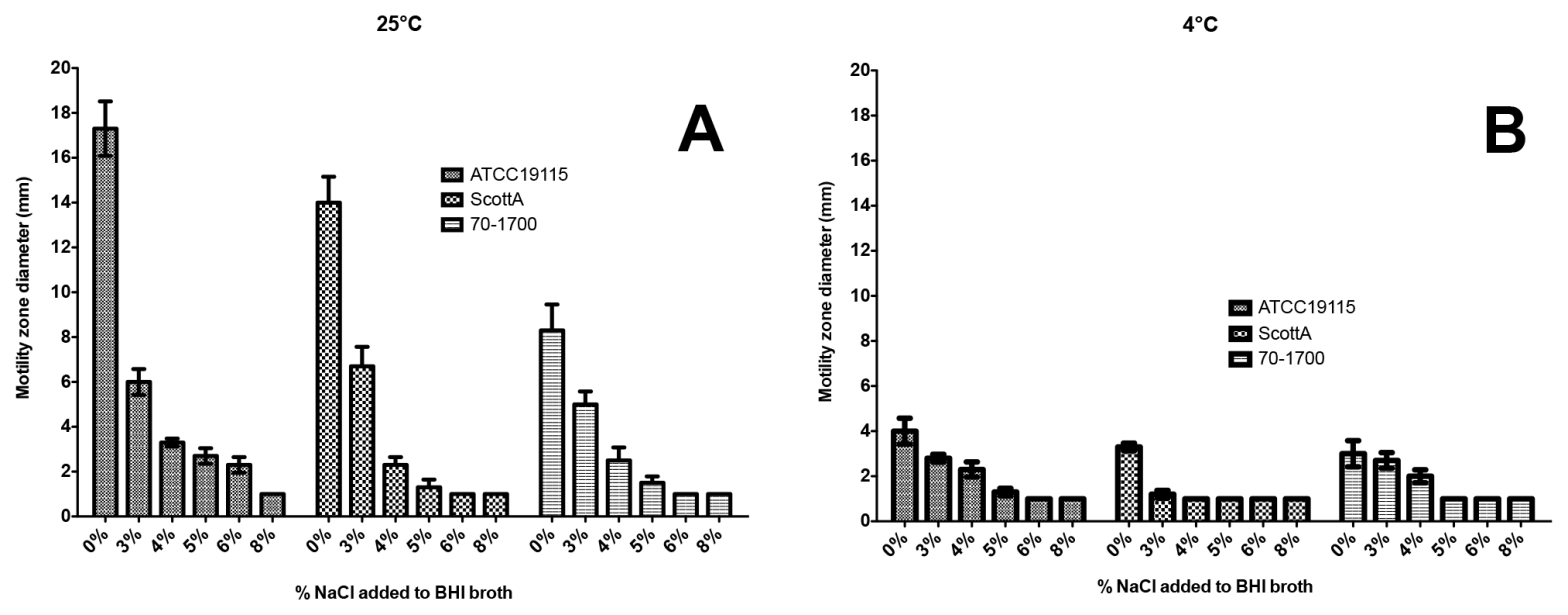
Swarming abilities of three strains of *L. monocytogenes* expressed as average motility zone diameters (mm). Swarming motility was assessed on semisoft BHIA plates supplemented with NaCl ranging from 0% to 8% w/v either a) at 25°C or b) at 4°C.

Increasing salt concentration and cold temperature had a strong inhibitory effect on the swarming motility of isolates tested (Figure 4). Addition of 3% NaCl to BHI medium resulted in a significant reduction of zone size in all strains examined, swarming motility ceased completely in BHI medium supplemented with 5% NaCl (Figure 4a). Loss of motility under osmotic stress has previously been reported in *E. coli*, which was correlated with a reduction in the amount of cellular flagellin [79]. Flagella have been shown to be an important factor in adherence to abiotic surfaces and subsequent biofilm formation [80,81] and cold temperature has been shown to decrease attachment of *L. monocytogenes* to various surfaces such as stainless steel [82] and polystyrene [83] although no direct assessment of motility has been evaluated in this organism upon exposure to environmental insult.

Flagella and flagellum-mediated motility are integral to the virulence of *L. monocytogenes* [84]. It was perhaps not surprising, then, that virulence related gene sets also showed suppression in stress adapted cells (Figure 3). Interestingly, cold temperature induced a stronger inhibition of virulence genes compared to hyperosmotic stress; suggesting that cold temperature is a stronger trigger for virulence suppression. This most likely is due to inherent recognition of low temperature as environmental cue far removed from those encountered within a mammalian host [85] and as such represents part of *L. monocytogenes* suite of saprophytic adaptations and is quite distinct from the parasitic adaptation relevant to host survival and listeriosis.

Overall, adaptation of *L. monocytogenes* to both hyperosmotic and cold stress is associated with decrease in the expression of cell surface proteins, flagella, and other virulence-related external proteins such as internalins (Tables S3, S5 and S7). The benefit in stripping of surface proteins in stressed cells is unclear, however might be influenced by the thickening and restructuring of the bacterial envelope associated with acclimatisation to the environment [58,86] and could be a general feature of *L. monocytogenes* in its saprophytic mode.

### Regulation of transcription during adaptation to hyperosmotic and cold stress

Survival of *L. monocytogenes* under non-host-associated environmental stress conditions such as osmotic and cold stresses has been attributed to tightly regulated gene transcription. Regulation of gene expression in *L. monocytogenes* consists of a complex network of interactions between different transcriptional regulators and alternative sigma factors [87]. The complexities of these interactions which contribute to fine-tuning gene expression under stress conditions are becoming more apparent with transcriptomic studies, thus the exact nature of these processes remains only partly understood.

**Figure 5:**
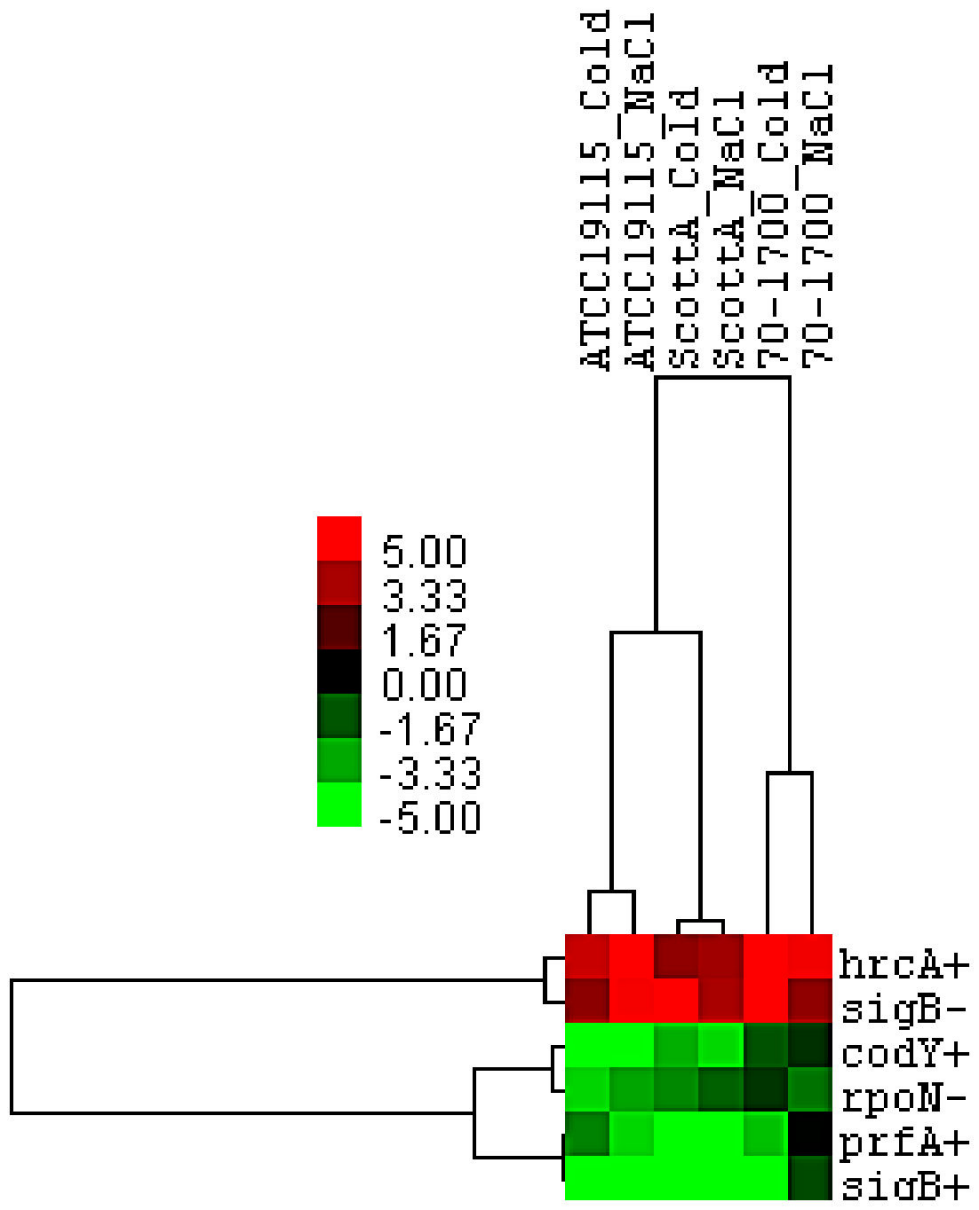
Heat map showing statistical trends for regulons in strains of *L. monocytogenes* exposed to either sub-lethal level of NaCl or cold stresses. T-test based procedure was used to score the changes in expression of each regulon, statistically significant T-values (as determined by a two-tailed P-value) are expressed from -5 (shown in green) to 5 (shown in red). Gene sets were considered significantly up-regulated with T-value ≥ 1 and significantly down-regulated with T-value ≤ -1.

*L. monocytogenes* adapted to hyperosmotic and cold stress has a suppressed sigma B (σ^B^) regulon, evident by the strong down regulation of genes positively influenced by this general-stress master regulator in either cold or osmotically-adapted cells (Figure 5). A number of genes either directly or indirectly activated by σ^B^ showed strong down-regulation in stress-adapted cells, such as *dapD* (*lmo0265*), in addition genes directly or indirectly repressed by σ^B^ showed significant up-regulation in stress-adapted strains. Some of the highly activated genes repressed by σ^B^ included *rpmI* (*lmo1784*), *oppC* (*lmo2194*) and *lmo2522* (Table 1).

Proteomic [88], transcriptomic [13,85,89] and phenotypic characterisation of σ^B^ null mutants had provided sufficient evidence that this alternative sigma factor categorically contributes to the ability of *L. monocytogenes* to survive and proliferate under various stress conditions encountered in non-host-associated environments. It appears that σ^B^ plays a more passive role in cells already adapted to a given stress, since σ^B^ transcription occurs within minutes of stress exposure [90] it could be speculated that active regulation of gene expression in stress-adapted cells is left to other components of the regulatory network.

PrfA regulon also showed repression in both cold and salt adapted cells (Figure 5), which is in agreement with the observed overall down-regulation of virulence genes in these cells and the overlap between the σ^B^ and of PrfA regulons [91].

Genes indirectly influenced by the transcriptional repressor of heat shock genes, HrcA which itself is also positively regulated by σ^B^ [25,91], were de-repressed in *L. monocytogenes* adapted to either hyperosmotic stress or low temperature (Figure 5). The majority of transcriptionally up-regulated genes activated by HrcA included those encoding ribosomal proteins, which showed an overall activation in cells adapted to both stresses. The exact involvement of this repressor in stress response of *L. monocytogenes* is unclear particularly since regulation of genes by these regulators is intertwined with that of the σ^B^ regulon [24,25,91].

Sigma factor σ^L^ (RpoN) has been shown to contribute to growth of *L. monocytogenes* at cold temperature [92] and hyperosmotic environments [93]. The σ^L^ regulon appeared to be involved at least indirectly, in regulation of transcription in osmotically and low temperature-adapted *L. monocytogenes* cells, with genes directly suppressed by RpoN being overall down-regulated (Figure 5). RpoN regulation contributes mostly to carbohydrate metabolism in *L. monocytogenes* EGD-e, by directly influencing PTS [94], which was overall suppressed in cells adapted to both stresses (Figure 3).

Adaptation to both stresses induced the CodY regulon evident by down-regulation of genes that have an increased expression in a *codY* null mutant [27] which are thought to be directly repressed by CodY. This GTP/isoleucine sensing regulator acts as a sensor of the energetic capacity and nutritional status of the cell [95], it suppresses genes associated with carbohydrate metabolism and transport, motility and chemotaxis [27] and showed significant down regulation in *L. monocytogenes* adapted to hyperosmotic and also cold stresses presented in this study (Figure 3) as well as acid stress [96]. Activation of CodY regulon in stress adapted cells suggests that cold temperature and hyperosmotic stress impose restrictions on intracellular energy availability, leading to suppression of some energy consuming processes such as flagella biosynthesis and unnecessary active uptake of nutrients, thus conserving energy.

## Summary and Conclusions

Overall adaptation to hyperosmotic and low temperature growth conditions in three *L. monocytogenes* strains were found to have many parallels, especially when examined using a statistically-based ontological approach. Evidence was presented for strong activation of genes associated with protein synthesis, in particular those coding proteins of the ribosome and involved directly in transcription. Genes associated with DNA maintenance; modification to cell envelope, and cell division were also up-regulated. On the other hand strong suppression of genes associated with carbohydrate metabolism and transport as well as flagella assembly was evident in stress adapted cells most likely due to energy preservation via CodY regulon. This suggests that adaptation to these adverse conditions engages similar mechanisms to cope with the induced stressful environments.

Hyperosmotic and cold stress factors, though quite different in terms of physiochemical stress on bacterial cells, revealed many parallels in terms of gene expression in three strains of *L. monocytogenes* studied. The results suggest that a broadly similar genetic regulatory mechanism operates in response to cold and hyperosmotic stresses.

This study is the first to assess the genetic bases of cold and hyperosmotic stress adaptation in multiple strains of *L. monocytogenes* and contributes to the understanding of stress physiology in this organism. Ongoing work is currently underway to evaluate global stress response in *L. monocytogenes* using a comprehensive proteomics approach to further elucidate stress physiology.

## Supporting Information

Table S1 **Generation times of isolates cultivated under conditions of hyperosmotic stress induced by supplementing BHIB with 12.5% w/v of NaCl or cold temperature of 4°C.** ^#^ Strains were grown at 25°C in 10mL BHIB supplemented with 12.5% w/v NaCl on a shaking incubator with transmittance being monitored at 600 nm until stationary phase was reached. Data obtained was analysed with LISREL (Scientific Software International, SSI) to solve for μmax (h^-1^) and maximum cell density and determine root mean square deviation (NG indicates lack of growth).
^*^ Strains were grown in 200 µL BHIB at 4°C in microtiter plates and change in absorbance was monitored using BioRad Benchmark microplate reader at 540 nm until stationary phase was reached. Data obtained was alalysed using DMFit software package (Institute of Food Research, IFR, UK).
(DOCX)

Table S2 **Log ratios of significantly up-regulated genes in *L. monocytogenes* strain ATCC19115 independently adapted to hyperosmotic stress induced by supplementing BHIB with 10% w/v salt or 4°C cold-temperature stress.** * Gene nomenclature used as per *L. monocytogenes* EGD-e genome. Gene homologs and predicted functions were obtained collectively from variety of sources including circulating literature and web based databases.
# LR: log ratio. Genes were considered significantly up-regulated with LR >1 which is equivocal of twofold up-regulation.
¥ Genes with P value >0.05 were not statistically significant and were excluded from this table.
(DOCX)

Table S3 **Log ratios of significantly down-regulated genes in *L. monocytogenes* strain ATCC19115 independently adapted to hyperosmotic stress induced by supplementing BHIB with 10% w/v salt or 4°C cold-temperature stress.** * Gene nomenclature used as per *L. monocytogenes* EGD-e genome. Gene homologs and predicted functions were obtained collectively from variety of sources including circulating literature and web based databases.
# LR: log ratio. Genes were considered significantly down-regulated with LR <-1 which is equivocal of twofold down-regulation.
¥ Genes with P value >0.05 were not statistically significant and were excluded from this table.
(DOCX)

Table S4 **Log ratios of significantly up-regulated genes in *L. monocytogenes* strain ScottA independently adapted to hyperosmotic stress induced by supplementing BHIB with 12% w/v salt or 4°C cold-temperature stress.** * Gene nomenclature used as per *L. monocytogenes* EGD-e genome. Gene homologs and predicted functions were obtained collectively from variety of sources including circulating literature and web based databases.
# LR: log ratio. Genes were considered significantly up-regulated with LR >1 which is equivocal of twofold up-regulation.
¥ Genes with P value >0.05 were not statistically significant and were excluded from this table.
(DOCX)

Table S5 **Log ratios of significantly down-regulated genes in *L. monocytogenes* strain ScottA independently adapted to hyperosmotic stress induced by supplementing BHIB with 12% w/v salt or 4°C cold-temperature stress.** * Gene nomenclature used as per *L. monocytogenes* EGD-e genome. Gene homologs and predicted functions were obtained collectively from variety of sources including circulating literature and web based databases.
# LR: log ratio. Genes were considered significantly down-regulated with LR <-1 which is equivocal of twofold down-regulation.
¥ Genes with P value >0.05 were not statistically significant and were excluded from this table.
(DOCX)

Table S6 **Log ratios of significantly up-regulated genes in *L. monocytogenes* strain 70-1700 independently adapted to hyperosmotic stress induced by supplementing BHIB with 8% w/v salt or 4°C cold-temperature stress.** * Gene nomenclature used as per *L. monocytogenes* EGD-e genome. Gene homologs and predicted functions were obtained collectively from variety of sources including circulating literature and web based databases.
# LR: log ratio. Genes were considered significantly up-regulated with LR >1 which is equivocal of twofold up-regulation.
¥ Genes with P value >0.05 were not statistically significant and were excluded from this table.
(DOCX)

Table S7 **Log ratios of significantly down-regulated genes in *L. monocytogenes* strain 70-1700 independently adapted to hyperosmotic stress induced by supplementing BHIB with 8% w/v salt or 4°C cold-temperature stress.** * Gene nomenclature used as per *L. monocytogenes* EGD-e genome. Gene homologs and predicted functions were obtained collectively from variety of sources including circulating literature and web based databases.
# LR: log ratio. Genes were considered significantly down-regulated with LR <-1 which is equivocal of twofold down-regulation.
¥ Genes with P value >0.05 were not statistically significant and were excluded from this table.
(DOCX)
