# Supplementary material for: Characterisation of the Transcriptomes of Genetically Diverse Listeria monocytogenes Exposed to Hyperosmotic and Low Temperature Conditions Reveal Global Stress-Adaptation Mechanisms
Source: PLoS One. 2013 Sep 4;8(9):e73603. doi: 10.1371/journal.pone.0073603 (PMC3762727; doi:10.1371/journal.pone.0073603)
Supplement: Table S1 — Generation times of isolates cultivated under conditions of hyperosmotic stress induced by supplementing BHIB with 12.5% w/v of NaCl or cold temperature of 4°C. # Strains were grown at 25°C in 10mL BHIB supplemented with 12.5% w/v NaCl on a shaking incubator with transmittance being monitored at 600 nm until stationary phase was reached. Data obtained was analysed with LISREL (Scientific Software International, SSI) to solve for μmax (h-1) and maximum cell density and determine root mean square deviation (NG indicates lack of growth). * Strains were grown in 200 µL BHIB at 4°C in microtiter plates and change in absorbance was monitored using BioRad Benchmark microplate reader at 540 nm until stationary phase was reached. Data obtained was alalysed using DMFit software package (Institute of Food Research, IFR, UK). (DOCX) [file pone.0073603.s001.docx]

| Strain | Salt GR (hrs)# | 95% confidence lower | 95% confidence upper | Cold GR (hrs)* | Standard error | Strain origin |
| --- | --- | --- | --- | --- | --- | --- |
| **20423** | **NG** | - | - | **21.49** | 1.03 | food/factory |
| **20425** | **NG** | - | - | **23.67** | 2.5 | food/factory |
| **20432** | **NG** | - | - | **24.75** | 2.13 | food/factory |
| **00P23755** | **NG** | - | - | **30.15** | 1.94 | clinical |
| **01P27654** | **NG** | - | - | **32.82** | 2.54 | clinical |
| **02P00638** | **NG** | - | - | **32.25** | 4.09 | clinical |
| **02P37678** | **NG** | - | - | **38.5** | 0.92 | clinical |
| **02P79545** | **NG** | - | - | **29.73** | 2.43 | clinical |
| **02P82412** | **NG** | - | - | **27.64** | 1.47 | clinical |
| **03P136964** | **NG** | - | - | **30.07** | 1.00 | clinical |
| **05P24111** | **NG** | - | - | **40.64** | 1.43 | clinical |
| **195-s-1-154** | **NG** | - | - | **18.22** | 0.38 | food/factory |
| **195-s-1-242** | **NG** | - | - | **36.13** | 2.31 | food/factory |
| **195-s-1-367** | **NG** | - | - | **23.21** | 1.05 | food/factory |
| **195-s-1-60** | **NG** | - | - | **21.61** | 1.02 | food/factory |
| **231-s-7-232** | **NG** | - | - | **19.71** | 0.81 | food/factory |
| **231-s-7-566** | **NG** | - | - | **26.94** | 1.13 | food/factory |
| **241-s-1-349** | **NG** | - | - | **22.09** | 1.02 | food/factory |
| **265-s-3-352** | **NG** | - | - | **17.67** | 0.94 | food/factory |
| **265-s-3-745** | **NG** | - | - | **17.19** | 1.37 | food/factory |
| **409-s-3-431** | **NG** | - | - | **21.72** | 0.91 | food/factory |
| **830-s-7-62** | **NG** | - | - | **20.77** | 1.26 | food/factory |
| **997-s-7-63** | **NG** | - | - | **23.75** | 1.48 | food/factory |
| **62-2853** | **NG** | - | - | **31.72** | 0.10 | animal |
| **62-4693** | **7.1** | 0.6 | 0.9 | **21.27** | 0.81 | animal |
| **63-5635** | **7.9** | 0.6 | 0.8 | **40.72** | 1.41 | animal |
| **64-0738** | **NG** | - | - | **23.38** | 1.69 | animal |
| **64-1495** | **NG** | - | - | **26.56** | 1.02 | animal |
| **64-2389** | **NG** | - | - | **28.73** | 2.80 | animal |
| **66-0755** | **9** | 0.3 | 0.3 | **19.6** | 1.29 | animal |
| **67-1759** | **8** | 0.5 | 0.5 | **18.29** | 1.25 | animal |
| **67-1786** | **15.5** | 1.3 | 1.6 | **42.02** | 0.47 | animal |
| **68-2169** | **6.2** | 0.6 | 0.6 | **18.6** | 1.22 | animal |
| **68-2528** | **10.1** | 0.8 | 0.9 | **25.29** | 1.16 | animal |
| **69-0577** | **6.9** | 0.7 | 0.9 | **21.95** | 1.12 | animal |
| **69-1363** | **5.4** | 0.8 | 1.1 | **27.5** | 1.61 | animal |
| **69-1793** | **6.2** | 0.8 | 1.1 | **31.35** | 1.33 | animal |
| **70-0249** | **6.5** | 0.8 | 1.2 | **24.88** | 2.04 | animal |
| **70-0378** | **5.7** | 0.6 | 0.7 | **27.61** | 2.40 | animal |
| **70-0421** | **14.2** | 1.9 | 2.6 | **25.21** | 2.91 | animal |
| **70-1700** | **NG** | - | - | **32.66** | 2.44 | animal |
| **70-2058** | **NG** | - | - | **34.99** | 1.74 | animal |
| **70-3167** | **6.7** | 0.6 | 0.7 | **26.26** | 2.71 | animal |
| **71-0563** | **NG** | - | - | **16.95** | 0.60 | animal |
| **71-0934** | **8.7** | 0.7 | 0.8 | **18.41** | 1.20 | animal |
| **71-3227** | **6.2** | 0.7 | 0.9 | **24.58** | 0.69 | clinical |
| **72-0039** | **6.1** | 0.5 | 0.7 | **15.99** | 0.15 | animal |
| **73-0336** | **6.5** | 0.8 | 1.1 | **47.08** | 1.24 | animal |
| **73-1801** | **NG** | - | - | **12.23** | 0.55 | animal |
| **74-0490** | **NG** | - | - | **19.24** | 0.77 | animal |
| **74-2395** | **6.1** | 0.8 | 0.9 | **17.97** | 0.44 | animal |
| **76-1854** | **NG** | - | - | **9.9** | 0.55 | animal |
| **76-2120/1** | **15.7** | 1.5 | 2 | **19.15** | 0.59 | animal |
| **77-2294** | **5.7** | 0.8 | 1.2 | **16.8** | 0.40 | animal |
| **77-4745** | **7.1** | 0.7 | 0.7 | **15.35** | 0.76 | animal |
| **78-0712** | **7.8** | 0.7 | 0.9 | **19.62** | 1.14 | animal |
| **78-1098** | **NG** | - | - | **16.76** | 0.96 | animal |
| **78-2183** | **NG** | - | - | **15.12** | 0.45 | animal |
| **78-2755** | **NG** | - | - | **20.06** | 0.78 | animal |
| **78-3565** | **9** | 1 | 1.3 | **20.9** | 1.99 | animal |
| **78-3636** | **7.1** | 0.6 | 0.9 | **13.94** | 1.30 | animal |
| **79-0430** | **6.6** | 0.6 | 0.7 | **21.21** | 1.32 | animal |
| **79-0869** | **6.9** | 0.9 | 1.1 | **20.77** | 1.07 | animal |
| **79-1828** | **6.6** | 0.6 | 0.6 | **13.84** | 1.55 | animal |
| **79-1994** | **7.6** | 1 | 1.2 | **26.26** | 0.84 | animal |
| **79-2048** | **6.9** | 0.4 | 0.3 | **18.2** | 1.63 | animal |
| **79-2336** | **NG** | - | - | **20.84** | 2.65 | animal |
| **79-2336/1** | **NG** | - | - | **10.65** | 0.40 | animal |
| **79-2336/16** | **NG** | - | - | **12.66** | 0.42 | animal |
| **79-2336/3** | **9.2** | 0.6 | 0.6 | **10.2** | 0.55 | animal |
| **79-2360** | **6.8** | 0.7 | 0.8 | **16.97** | 0.49 | animal |
| **79-2759** | **7** | 0.6 | 0.7 | **18.58** | 1.11 | animal |
| **79-3194** | **6.4** | 0.7 | 0.7 | **19.95** | 1.67 | animal |
| **80-0619** | **7.3** | 0.6 | 0.7 | **16.97** | 1.25 | animal |
| **80-0910** | **12.2** | 1.4 | 1.6 | **24.58** | 1.07 | animal |
| **80-2437** | **7** | 0.5 | 0.6 | **19.96** | 0.39 | animal |
| **80-2880** | **6.5** | 0.4 | 0.5 | **24.02** | 0.53 | animal |
| **80-2901** | **6.5** | 0.5 | 0.5 | **25.61** | 1.42 | animal |
| **80-2942** | **6.3** | 0.8 | 1 | **16.68** | 0.40 | animal |
| **80-3354** | **11** | 1.2 | 1.7 | **16.41** | 1.05 | animal |
| **80-3453** | **NG** | - | - | **23.31** | 1.31 | animal |
| **80-3554** | **NG** | - | - | **21.77** | 0.18 | animal |
| **80-3749** | **7** | 0.5 | 0.5 | **24.56** | 3.39 | animal |
| **80-4762** | **6.8** | 0.6 | 0.7 | **22.32** | 0.40 | animal |
| **80-4798** | **7.5** | 0.7 | 1 | **18.16** | 0.45 | animal |
| **80-4904** | **7.5** | 0.7 | 1 | **15.55** | 0.36 | animal |
| **83-0159** | **7.4** | 0.4 | 0.5 | **18.9** | 1.03 | animal |
| **83-1617** | **9.6** | 1 | 1.1 | **16.71** | 0.38 | animal |
| **83-1804** | **7.6** | 0.9 | 1.2 | **16.89** | 2.19 | animal |
| **83-1885** | **8** | 0.5 | 0.5 | **17.43** | 2.09 | animal |
| **83-2099** | **NG** | - | - | **16.12** | 0.43 | animal |
| **83-2795** | **6.9** | 0.5 | 0.7 | **19.49** | 2.58 | animal |
| **84-1886** | **5.9** | 0.7 | 1.1 | **18.71** | 1.17 | animal |
| **84-2026** | **7.2** | 0.6 | 0.7 | **16.15** | 1.98 | animal |
| **85-0010** | **6.7** | 0.5 | 0.6 | **18.86** | 0.89 | animal |
| **85-0567** | **7.8** | 0.5 | 0.6 | **15.73** | 1.02 | animal |
| **85-0658** | **6.2** | 0.6 | 0.8 | **20.74** | 0.89 | animal |
| **85-2389** | **6.5** | 0.9 | 1.3 | **19.67** | 0.84 | animal |
| **86-0071** | **6.4** | 0.6 | 0.7 | **15.17** | 0.47 | animal |
| **86-3009** | **NG** | - | - | **14.27** | 0.55 | animal |
| **87-0041** | **7.6** | 0.7 | 0.9 | **14.4** | 0.60 | animal |
| **87-0707** | **6.6** | 0.3 | 0.4 | **14.47** | 1.12 | animal |
| **87-1599** | **6.9** | 0.7 | 0.9 | **14.75** | 0.57 | animal |
| **87-2555** | **7.2** | 1.2 | 1.8 | **16.82** | 0.75 | animal |
| **89-1931** | **NG** | - | - | **15.11** | 0.70 | animal |
| **90-0053** | **NG** | - | - | **19.79** | 1.15 | animal |
| **91B3450** | **NG** | - | - | **37.6** | 2.42 | clinical |
| **92-0305** | **6.5** | 0.6 | 0.7 | **22.88** | 2.43 | unknown |
| **97P107857** | **NG** | - | - | **26.4** | 2.92 | clinical |
| **98P130512** | **NG** | - | - | **30.8** | 2.05 | clinical |
| **98P1437** | **NG** | - | - | **17.08** | 1.27 | clinical |
| **99P154255** | **NG** | - | - | **35.19** | 0.84 | clinical |
| **99P63881** | **NG** | - | - | **27.51** | 0.63 | clinical |
| **ATCC 10403** | **7.6** | 0.5 | 0.7 | **19.48** | 2.36 | clinical |
| **ATCC 19111** | **NG** | - | - | **31.42** | 2.34 | clinical |
| **ATCC 19112** | **NG** | - | - | **19.1** | 1.48 | clinical |
| **ATCC 19114** | **NG** | - | - | **21.62** | 1.14 | animal |
| **ATCC 19115** | **NG** | - | - | **23.86** | 1.19 | clinical |
| **ATCC 33090** | **NG** | - | - | **27.87** | 1.33 | animal |
| **ATCC 7644** | **NG** | - | - | **18.91** | 0.39 | clinical |
| **FRRW 2343** | **NG** | - | - | **20.49** | 1.05 | food/factory |
| **FRRW 2345** | **NG** | - | - | **22.06** | 0.66 | food/factory |
| **FW03/0032** | **8.5** | 0.9 | 1 | **15.46** | 0.38 | food/factory |
| **FW03/0033** | **7.4** | 0.8 | 1.1 | **17.56** | 0.99 | food/factory |
| **FW03/0034** | **9.4** | 1.5 | 2.1 | **29.52** | 0.96 | food/factory |
| **FW03/0035** | **5.3** | 0.4 | 0.4 | **30.18** | 3.38 | food/factory |
| **FW03/0036** | **8.3** | 0.4 | 0.4 | **14.11** | 0.56 | food/factory |
| **FW04/0017** | **11.2** | 1.5 | 2 | **19.21** | 0.37 | clinical |
| **FW04/0018** | **12.5** | 0.7 | 0.8 | **23.51** | 0.55 | clinical |
| **FW04/0019** | **10.7** | 1.3 | 1.7 | **29.66** | 1.35 | clinical |
| **FW04/0020** | **5.9** | 1 | 1.5 | **15.32** | 1.21 | clinical |
| **FW04/0021** | **5.9** | 0.6 | 0.8 | **15.45** | 1.29 | clinical |
| **FW04/0022** | **11.1** | 1.3 | 1.6 | **21.32** | 0.71 | food/factory |
| **FW04/0023** | **6.9** | 0.7 | 0.9 | **24.39** | 0.89 | food/factory |
| **FW04/0024** | **9** | 0.6 | 0.6 | **15.37** | 1.39 | food/factory |
| **FW04/0025** | **NG** | - | - | **23.25** | 2.53 | food/factory |
| **FW04/0026** | **12** | 0.8 | 0.9 | **23.67** | 1.40 | food/factory |
| **FW04/0037** | **6.2** | 0.6 | 0.7 | **13.17** | 0.50 | food/factory |
| **Joyce** | **11.5** | 0.7 | 0.8 | **29.69** | 2.17 | animal |
| **L1** | **6.5** | 0.4 | 0.5 | **19.09** | 1.34 | unknown |
| **L2** | **10.1** | 1.1 | 1.5 | **18.69** | 0.22 | unknown |
| **L522** | **NG** | - | - | **24.14** | 1.36 | food/factory |
| **LM 412** | **NG** | - | - | **21.95** | 0.8 | unknown |
| **LM 412N** | **NG** | - | - | **23.61** | 1.87 | unknown |
| **LM NR30** | **NG** | - | - | **21.3** | 0.62 | unknown |
| **LO28** | **14.1** | 1.2 | 1.4 | **26.26** | 1.33 | clinical |
| **S204231_1** | **10.5** | 1.2 | 1.5 | **19.63** | 1.90 | food/factory |
| **ScottA** | **6.7** | 0.6 | 0.7 | **32.28** | 1.34 | clinical |
