# Supplementary material for: Characterisation of the Transcriptomes of Genetically Diverse Listeria monocytogenes Exposed to Hyperosmotic and Low Temperature Conditions Reveal Global Stress-Adaptation Mechanisms
Source: PLoS One. 2013 Sep 4;8(9):e73603. doi: 10.1371/journal.pone.0073603 (PMC3762727; doi:10.1371/journal.pone.0073603)
Supplement: Table S2 — Log ratios of significantly up-regulated genes in L. monocytogenes strain ATCC19115 independently adapted to hyperosmotic stress induced by supplementing BHIB with 10% w/v salt or 4°C cold-temperature stress. * Gene nomenclature used as per L. monocytogenes EGD-e genome. Gene homologs and predicted functions were obtained collectively from variety of sources including circulating literature and web based databases. # LR: log ratio. Genes were considered significantly up-regulated with LR >1 which is equivocal of twofold up-regulation. ¥ Genes with P value >0.05 were not statistically significant and were excluded from this table. (DOCX) [file pone.0073603.s002.docx]

| Gene^*^ | Salt adapted | | Cold adapted | | Function |
| --- | --- | --- | --- | --- | --- |
|  | LR^#^ | P^¥^ | LR | P |  |
| *dnaA* | **1.89** | 0.001 | **1.53** | 0.003 | chromosomal replication initiator protein |
| *lmo0010* | **3.98** | 0.000 | **1.07** | 0.005 | mevalonate kinase |
| *rpsF* | **1.97** | 0.000 | **1.34** | 0.003 | ribosomal protein S6 |
| *ssb* | **3.32** | 0.001 | **1.47** | 0.009 | single-stranded DNA-binding protein |
| *rpsR* | **1.66** | 0.000 | **1.20** | 0.021 | ribosomal protein S18 |
| *lmo0047* | **1.57** | 0.012 | **2.12** | 0.033 | predicted membrane protein |
| *purA* | **1.26** | 0.037 | **1.76** | 0.005 | adenylosuccinate synthetase |
| *holB* | **2.38** | 0.000 | **2.14** | 0.000 | DNA polymerase III delta' subunit |
| *metS* | **1.08** | 0.023 | **1.12** | 0.005 | methionyl-tRNA synthetase |
| *lmo0189* | **3.31** | 0.000 | **5.28** | 0.000 | similar to uncharacterized conserved proteins; similar to B. subtilis Veg protein |
| *ispE* | **2.14** | 0.002 | **1.20** | 0.018 | 4-diphosphocytidyl-2-C-methyl-D-erythritol kinase |
| *lmo0191* | **1.70** | 0.002 | **1.49** | 0.023 | similar to a putative phospho-beta-glucosidase |
| *acrA* | **1.28** | 0.041 | **2.44** | 0.000 | putative HlyD family secretion protein; membrane fusion protein |
| *lmo0194* | **1.14** | 0.006 | **2.38** | 0.000 | similar to ABC transporter, ATP-binding protein |
| *lmo0196* | **2.28** | 0.000 | **2.60** | 0.001 | similar to uncharacterized protein involved in the regulation of septum location |
| *spoVG* | **2.15** | 0.000 | **1.95** | 0.001 | similar to uncharacterized protein involved in the regulation of septum location |
| *gcaD* | **2.10** | 0.001 | **2.35** | 0.000 | glucosamine-1-phosphate N-acetyltransferase / UDP-N-acetylglucosamine pyrophosphorylase |
| *prs* | **2.28** | 0.008 | **1.62** | 0.002 | phosphoribosyl pyrophosphate synthetase |
| *lmo0217* | **1.19** | 0.010 | **1.77** | 0.000 | similar to septum formation initiators |
| *lmo0222* | **1.02** | 0.016 | **1.01** | 0.002 | chaperonin (heat shock protein 33) |
| *lmo0227* | **3.21** | 0.000 | **1.88** | 0.001 | putative tRNA-dihydrouridine |
| *rpmG* | **1.16** | 0.008 | **1.79** | 0.000 | ribosomal protein L33 |
| *secE* | **2.67** | 0.004 | **3.12** | 0.000 | preprotein translocase SecE subunit |
| *nusG* | **1.49** | 0.003 | **2.19** | 0.000 | transcription antitermination factor |
| *rplK* | **3.36** | 0.001 | **2.25** | 0.000 | ribosomal protein L11 |
| *rplA* | **3.37** | 0.001 | **1.71** | 0.002 | ribosomal protein L1 |
| *lmo0289* | **1.78** | 0.001 | **1.46** | 0.001 | similar to uncharacterized conserved proteins |
| *lmo0581* | **1.35** | 0.020 | **1.23** | 0.006 | similar to predicted SAM-dependent methyltransferases |
| *lmo0763* | **1.65** | 0.003 | **1.18** | 0.001 | similar to predicted phosphohydrolases |
| *lmo0857* | **1.39** | 0.024 | **1.21** | 0.003 | similar to esterase/lipase family proteins |
| *lmo0957* | **1.42** | 0.041 | **1.05** | 0.037 | glucosamine-6 phosphate isomerase |
| *htpX* | **1.03** | 0.016 | **1.53** | 0.002 | similar to putative heat shock protein HtpX; Listeria epitope LemB |
| *lmo1008* | **1.25** | 0.009 | **2.15** | 0.001 | unknown protein |
| *pycA* | **1.56** | 0.004 | **2.69** | 0.000 | pyruvate carboxylase |
| *guaA* | **1.09** | 0.016 | **1.15** | 0.001 | GMP synthase (glutamine hydrolyzing) |
| *eutH* | **1.40** | 0.004 | **1.22** | 0.001 | similar to ethanolamine utilisation EutH protein (putative periplasmic transport protein) |
| *trxA* | **2.09** | 0.039 | **1.53** | 0.002 | thioredoxin |
| *racE* | **1.45** | 0.003 | **1.15** | 0.002 | glutamate racemase |
| *lmo1238* | **1.40** | 0.016 | **1.53** | 0.002 | similar to ribonuclease PH |
| *lmo1245* | **1.57** | 0.007 | **1.41** | 0.000 | unknown protein |
| *tig* | **1.82** | 0.001 | **2.21** | 0.004 | trigger factor (prolyl isomerase) |
| *lmo1271* | **1.33** | 0.016 | **1.62** | 0.001 | signal peptidase I |
| *topA* | **1.36** | 0.001 | **1.47** | 0.006 | topoisomerase IA |
| *lmo1282* | **1.83** | 0.043 | **1.18** | 0.007 | unknown protein |
| *lmo1306* | **3.18** | 0.005 | **3.80** | 0.000 | similar to uncharacterized conserved proteins |
| *lmo1315* | **1.07** | 0.010 | **2.41** | 0.000 | undecaprenyl pyrophosphate synthase |
| *lmo1317* | **1.26** | 0.009 | **1.15** | 0.001 | 1-deoxy-D-xylulose-5-phosphate reductoisomerase |
| *lmo1318* | **2.95** | 0.000 | **1.33** | 0.002 | putative membrane-associated Zn-dependent metalloprotease |
| *proS* | **1.22** | 0.011 | **1.03** | 0.004 | prolyl-tRNA synthetase |
| *lmo1323* | **2.03** | 0.003 | **1.10** | 0.002 | similar to predicted nucleic-acid-binding protein implicated in transcription termination |
| *pnpA* | **1.90** | 0.001 | **1.34** | 0.000 | polyribonucleotide nucleotidyltransferase |
| *lmo1333* | **1.71** | 0.008 | **4.21** | 0.000 | similar to aminodeoxychorismate lyase family proteins |
| *lmo1337* | **1.96** | 0.007 | **2.52** | 0.000 | uncharacterized membrane protein |
| *lmo1339* | **1.79** | 0.008 | **1.59** | 0.001 | glucokinase |
| *cspL* | **2.85** | 0.000 | **2.77** | 0.010 | similar to cold shock protein (beta-ribbon, CspA family) |
| *lmo1414* | **1.16** | 0.009 | **1.62** | 0.001 | acetyl-CoA acetyltransferase |
| *lmo1431* | **2.67** | 0.000 | **3.06** | 0.000 | ABC transporter, ATP-binding protein |
| *lmo1436* | **1.41** | 0.008 | **1.10** | 0.039 | similar to aspartokinase I (alpha and beta subunits) |
| *zurR* | **1.09** | 0.039 | **1.08** | 0.005 | zinc uptake regulator |
| *lmo1448* | **1.15** | 0.008 | **1.27** | 0.023 | inorganic pyrophosphatase/exopolyphosphatase |
| *rpsU* | **1.56** | 0.000 | **1.35** | 0.013 | ribosomal protein S21 |
| *greA* | **1.28** | 0.012 | **2.16** | 0.000 | transcription elongation factor |
| *lmo1511* | **1.45** | 0.006 | **1.27** | 0.001 | similar to lysophospholipase family proteins and other enzymes with an alpha/beta hydrolase fold |
| *hisS* | **1.15** | 0.019 | **1.21** | 0.001 | histidyl-tRNA synthetase |
| *rpmA* | **1.31** | 0.003 | **2.20** | 0.012 | ribosomal protein L27 |
| *lmo1541* | **2.33** | 0.001 | **2.60** | 0.003 | predicted ribosomal protein |
| *rplU* | **2.74** | 0.000 | **2.90** | 0.005 | ribosomal protein L21 |
| *lmo1582* | **2.13** | 0.001 | **2.23** | 0.000 | putative adenine-specific DNA methylase |
| *rpsD* | **3.21** | 0.001 | **1.32** | 0.001 | ribosomal protein S4 |
| *aroA* | **1.43** | 0.009 | **1.97** | 0.000 | phospho-2-dehydro-3-deoxyheptonate aldolase / chorismate mutase |
| *lmo1626* | **1.21** | 0.014 | **2.02** | 0.000 | unknown protein |
| *lmo1636* | **1.40** | 0.010 | **1.18** | 0.001 | similar to ABC transporter, ATP-binding protein |
| *rpsB* | **2.22** | 0.000 | **2.34** | 0.000 | ribosomal protein S2 |
| *lmo1707* | **1.95** | 0.000 | **1.56** | 0.008 | unknown protein |
| *lmo1709* | **1.60** | 0.019 | **1.13** | 0.001 | simular to methionyl aminopeptidase |
| *lmo1743* | **1.40** | 0.008 | **1.36** | 0.004 | unknown protein |
| *lmo1744* | **1.13** | 0.019 | **1.06** | 0.002 | putative nucleoside-diphosphate-sugar epimerases |
| *lmo1760* | **2.18** | 0.001 | **1.97** | 0.000 | predicted phosphate-binding enzyme |
| *rpmI* | **2.39** | 0.000 | **1.32** | 0.001 | ribosomal protein L35 |
| *infC* | **1.30** | 0.019 | **2.15** | 0.000 | translation initiation factor IF3 |
| *rplS* | **1.94** | 0.001 | **1.88** | 0.001 | ribosomal protein L19 |
| *acpA* | **1.36** | 0.005 | **3.58** | 0.002 | acyl carrier protein |
| *rpmB* | **3.27** | 0.000 | **4.34** | 0.001 | ribosomal protein L28 |
| *lmo1874* | **1.04** | 0.022 | **1.12** | 0.001 | thymidylate synthase |
| *recU* | **1.31** | 0.014 | **1.33** | 0.010 | similar to DNA repair and homologous recombination protein |
| *panB* | **2.38** | 0.001 | **1.05** | 0.003 | ketopantoate hydroxymethyltransferase |
| *lmo1914* | **1.58** | 0.033 | **1.39** | 0.026 | similar to predicted sensor kinases |
| *lmo1921* | **2.13** | 0.004 | **1.44** | 0.004 | unknown protein |
| *lmo1922* | **1.74** | 0.006 | **1.32** | 0.007 | similar to pilus assembly protein |
| *menH* | **1.03** | 0.013 | **1.04** | 0.014 | 2-heptaprenyl-1,4-naphthoquinone methyltransferase |
| *rpsA* | **1.00** | 0.025 | **1.61** | 0.008 | ribosomal protein S1 |
| *lmo1941* | **1.56** | 0.003 | **2.16** | 0.000 | similar to uncharacterized conserved proteins |
| *resD* | **1.93** | 0.002 | **1.97** | 0.001 | two-component response regulator |
| *lmo1950* | **2.10** | 0.001 | **1.14** | 0.001 | segregation and condensation protein B |
| *lysA* | **1.26** | 0.007 | **1.00** | 0.002 | diaminopimelate decarboxylase |
| *xerD* | **1.01** | 0.011 | **1.66** | 0.000 | similar to integrase/recombinase |
| *fhuC* | **1.57** | 0.003 | **1.02** | 0.002 | similar to ferrichrome ABC transporter, ATP binding protein |
| *lmo1978* | **1.48** | 0.008 | **1.47** | 0.000 | glucose-6-phosphate 1-dehydrogenase |
| *lmo1979* | **2.07** | 0.001 | **1.06** | 0.002 | similar to uncharacterized conserved proteins |
| *divIVA* | **1.14** | 0.029 | **2.02** | 0.001 | similar to cell-division initiation protein (septum placement) |
| *murG* | **1.36** | 0.004 | **1.51** | 0.004 | UDP-N-acetylglucosamine-N-acetylmuramyl-(pentapeptide) pyrophosphoryl-undecaprenol N-acetylglucosamine transferase |
| *lmo2048* | **2.70** | 0.000 | **1.15** | 0.003 | similar to uncharacterized conserved proteins |
| *lmo2056* | **1.43** | 0.004 | **1.47** | 0.000 | similar to uncharacterized conserved proteins |
| *lmo2071* | **1.69** | 0.001 | **1.13** | 0.001 | Unknown protein |
| *lmo2075* | **2.21** | 0.000 | **1.05** | 0.002 | similar to O-sialoglycoprotein endopeptidase |
| *lmo2117* | **1.11** | 0.028 | **1.15** | 0.001 | similar to acetyltransferase (GNAT) family proteins |
| *oppE* | **2.11** | 0.001 | **1.18** | 0.054 | similar to oligopeptide ABC transporter, ATP binding protein |
| *oppC* | **1.44** | 0.003 | **1.82** | 0.022 | similar to oligopeptide ABC transporter, permease protein |
| *oppB* | **1.62** | 0.003 | **1.63** | 0.001 | similar to oligopeptide ABC transporter, permease protein |
| *lmo2197* | **1.69** | 0.002 | **1.01** | 0.007 | unknown protein |
| *lmo2201* | **2.66** | 0.003 | **1.71** | 0.000 | 3-oxoacyl-[acyl-carrier-protein] synthase I/II |
| *lmo2202* | **1.53** | 0.003 | **1.96** | 0.000 | 3-oxoacyl-[acyl-carrier-protein] synthase III |
| *lmo2208* | **1.20** | 0.018 | **1.20** | 0.007 | similar to predicted hydrolases of the HAD superfamily |
| *hemE* | **1.16** | 0.017 | **1.09** | 0.041 | uroporphyrinogen III decarboxylase |
| *prsA* | **2.81** | 0.000 | **5.79** | 0.000 | similar to post-translocation molecular chaperone |
| *lmo2223* | **2.41** | 0.000 | **2.28** | 0.003 | similar to uncharacterized conserved proteins |
| *lmo2241* | **2.92** | 0.001 | **1.52** | 0.001 | similar to transcription regulator, GntR family |
| *lmo2247* | **1.21** | 0.033 | **1.50** | 0.000 | similar to aldo/keto reductases, related to diketogulonate reductase |
| *lmo2249* | **1.08** | 0.014 | **1.62** | 0.000 | similar to low-affinity inorganic phosphate transporter |
| *fruA* | **1.12** | 0.007 | **1.24** | 0.006 | similar to PTS system, fructose-specific IIABC component |
| *lmo2359* | **1.71** | 0.001 | **3.34** | 0.000 | similar to predicted hydrolases of the HAD superfamily |
| *lmo2376* | **1.92** | 0.009 | **1.96** | 0.000 | similar to peptidyl-prolyl cis-trans isomerase |
| *lmo2407* | **1.50** | 0.005 | **4.69** | 0.000 | unknown protein |
| *lmo2420* | **1.52** | 0.004 | **1.10** | 0.005 | Unknown protein |
| *lmo2422* | **1.05** | 0.054 | **1.89** | 0.034 | similar to two-component response regulator |
| *lmo2428* | **2.02** | 0.001 | **1.64** | 0.000 | similar to FtsK/RodA/SpoIIIE and related proteins |
| *cggR* | **4.32** | 0.000 | **3.43** | 0.000 | central glycolytic genes regulator |
| *lmo2479* | **1.44** | 0.054 | **1.13** | 0.045 | similar to uncharacterized conserved proteins |
| *lmo2503* | **1.17** | 0.020 | **1.47** | 0.002 | similar to cardiolipin synthase |
| *lmo2504* | **2.46** | 0.000 | **2.65** | 0.000 | similar to membrane-bound metallopeptidases |
| *ftsE* | **2.34** | 0.000 | **2.66** | 0.000 | cell division protein; ABC Transporter, permease protein |
| *ftsX* | **5.92** | 0.000 | **1.02** | 0.012 | cell division protein; ABC Transporter, ATP-binding protein |
| *lmo2508* | **1.67** | 0.001 | **1.84** | 0.000 | similar to uncharacterized conserved proteins |
| *lmo2522* | **2.11** | 0.009 | **6.55** | 0.000 | similar to uncharacterized conserved proteins |
| *atpA* | **1.42** | 0.002 | **1.71** | 0.008 | F0F1-type ATP synthase, alpha subunit |
| *atpH* | **1.43** | 0.002 | **1.59** | 0.031 | F0F1-type ATP synthase, delta subunit |
| *hom* | **2.50** | 0.000 | **1.71** | 0.000 | homoserine dehydrogenase |
| *rpmE* | **2.59** | 0.000 | **2.49** | 0.001 | ribosomal protein L31 |
| *lmo2555* | **1.68** | 0.001 | **2.55** | 0.000 | similar to glycosyltransferases |
| *fbaA* | **2.41** | 0.000 | **2.60** | 0.005 | fructose-bisphosphate aldolase |
| *lmo2563* | **1.03** | 0.019 | **1.04** | 0.001 | similar to Zn-dependent protease SpoIVFB |
| *rpmD* | **2.44** | 0.000 | **2.21** | 0.003 | ribosomal protein L30 |
| *rpsJ* | **2.07** | 0.002 | **1.05** | 0.006 | ribosomal protein S10 |
| *lmo2638* | **1.19** | 0.035 | **1.17** | 0.003 | NADH dehydrogenase |
| *fus* | **2.28** | 0.000 | **1.16** | 0.001 | elongation factor EF-G |
| *rpsG* | **3.41** | 0.000 | **1.39** | 0.010 | ribosomal protein S7 |
| *rpsL* | **4.10** | 0.000 | **2.06** | 0.000 | ribosomal protein S12 |
| *lmo2703* | **1.22** | 0.018 | **1.26** | 0.012 | similar to uncharacterized conserved proteins |
| *lmo2843* | **2.47** | 0.000 | **1.17** | 0.004 | similar to uncharacterized protein involved in cytokinesis |
| *lmo2857* | **1.19** | 0.034 | **1.71** | 0.000 | unknown protein |
