# Supplementary material for: Characterisation of the Transcriptomes of Genetically Diverse Listeria monocytogenes Exposed to Hyperosmotic and Low Temperature Conditions Reveal Global Stress-Adaptation Mechanisms
Source: PLoS One. 2013 Sep 4;8(9):e73603. doi: 10.1371/journal.pone.0073603 (PMC3762727; doi:10.1371/journal.pone.0073603)
Supplement: Table S3 — Log ratios of significantly down-regulated genes in L. monocytogenes strain ATCC19115 independently adapted to hyperosmotic stress induced by supplementing BHIB with 10% w/v salt or 4°C cold-temperature stress. * Gene nomenclature used as per L. monocytogenes EGD-e genome. Gene homologs and predicted functions were obtained collectively from variety of sources including circulating literature and web based databases. # LR: log ratio. Genes were considered significantly down-regulated with LR <-1 which is equivocal of twofold down-regulation. ¥ Genes with P value >0.05 were not statistically significant and were excluded from this table. (DOCX) [file pone.0073603.s003.docx]

| Gene^*^ | Salt adapted | | Cold adapted | | Function |
| --- | --- | --- | --- | --- | --- |
|  | LR^#^ | P^¥^ | LR | P |  |
| *lmo0027* | **-1.52** | 0.008 | **-2.11** | 0.000 | similar to PTS system, beta-glucosides-specific IIABC component |
| *lmo0033* | **-1.38** | 0.009 | **-1.19** | 0.001 | putative endoglucanase/glycosyl hydrolase |
| *lmo0036* | **-1.91** | 0.006 | **-2.91** | 0.000 | ornithine carbamoyltransferase |
| *lmo0038* | **-1.60** | 0.012 | **-2.38** | 0.000 | similar to peptidylarginine deiminase and related enzymes |
| *lmo0063* | **-1.25** | 0.035 | **-1.14** | 0.004 | unknown protein |
| *lmo0075* | **-1.80** | 0.001 | **-1.05** | 0.013 | similar to phosphoenolpyruvate phosphonomutase and related enzymes |
| *lmo0076* | **-1.19** | 0.012 | **-1.35** | 0.010 | methylated-DNA-[protein]-cysteine S-methyltransferase |
| *lmo0079* | **-1.53** | 0.003 | **-1.27** | 0.004 | unknown protein |
| *lmo0086* | **-1.48** | 0.019 | **-1.22** | 0.010 | unknown protein |
| *lmaA* | **-1.25** | 0.020 | **-2.91** | 0.000 | predicted secreted protein; Antigen A protein |
| *lmo0120* | **-1.27** | 0.028 | **-2.58** | 0.000 | similar to phage proteins |
| *lmo0124* | **-1.37** | 0.013 | **-2.35** | 0.000 | unknown protein |
| *lmo0127* | **-1.25** | 0.013 | **-1.26** | 0.004 | similar to protein gp20 from Bacteriophage A118 |
| *lmo0128* | **-1.39** | 0.022 | **-2.19** | 0.000 | similar to a holin protein from Bacteriophage phi-105 |
| *lmo0181* | **-2.22** | 0.000 | **-1.04** | 0.006 | similar to mutiple sugar ABC transporter, substrate binding protein |
| *lmo0234* | **-1.10** | 0.023 | **-1.19** | 0.009 | putative ntegral membrane protein |
| *lmo0261* | **-1.49** | 0.008 | **-1.58** | 0.000 | similar to phospho-beta-glucosidase |
| *lmo0265* | **-3.11** | 0.002 | **-2.03** | 0.002 | putative succinyl-diaminopimelate desuccinylase |
| *lmo0314* | **-1.43** | 0.005 | **-1.21** | 0.001 | similar to uncharacterized conserved proteins |
| *tenA* | **-1.30** | 0.008 | **-1.32** | 0.003 | similar to putative transcription activator of thiamine biosynthesis operon |
| *lmo0316* | **-2.02** | 0.000 | **-1.31** | 0.003 | hydroxyethylthiazole kinase |
| *lmo0345* | **-1.54** | 0.008 | **-1.40** | 0.008 | similar to ribose 5-phosphate isomerase B |
| *lmo0346* | **-1.26** | 0.004 | **-1.69** | 0.019 | similar to triosephosphate isomerase |
| *lmo0348* | **-1.10** | 0.031 | **-1.64** | 0.003 | similar to dihydroxyacetone kinase, N-terminal domain |
| *lmo0353* | **-1.08** | 0.014 | **-1.10** | 0.012 | similar to acetyltransferases |
| *lmo0377* | **-1.43** | 0.008 | **-1.10** | 0.005 | Unknown protein |
| *lmo0401* | **-3.69** | 0.000 | **-3.25** | 0.000 | putative alpha-mannosidase |
| *lmo0402* | **-2.27** | 0.001 | **-2.06** | 0.002 | fructose-specific PTS system operon regulator |
| *inlA* | **-1.98** | 0.002 | **-1.89** | 0.000 | internalin A |
| *lmo0505* | **-1.11** | 0.019 | **-1.27** | 0.000 | similar to ribulose-5-phosphate 3 epimerase |
| *lmo0515* | **-3.68** | 0.000 | **-3.68** | 0.000 | similar to universal stress protein UspA and related nucleotide-binding proteins |
| *lmo0546* | **-1.06** | 0.039 | **-1.18** | 0.011 | oxidoreductase family protein |
| *hisJ* | **-1.03** | 0.012 | **-1.46** | 0.004 | histidinol-phosphatase |
| *lmo0639* | **-1.01** | 0.017 | **-1.39** | 0.002 | similar to surface protein PAg negative regulator par |
| *lmo0643* | **-1.98** | 0.007 | **-2.00** | 0.001 | similar to transaldolase-like proteins |
| *lmo0659* | **-1.93** | 0.010 | **-1.18** | 0.003 | similar to predicted transcriptional regulators |
| *lmo0684* | **-1.21** | 0.049 | **-1.72** | 0.017 | unknown protein |
| *lmo0687* | **-1.05** | 0.018 | **-1.70** | 0.002 | Unknown protein |
| *flaA* | **-3.43** | 0.000 | **-4.09** | 0.000 | flagellin |
| *lmo0694* | **-1.64** | 0.001 | **-1.43** | 0.000 | unknown protein |
| *flgE* | **-1.01** | 0.015 | **-1.96** | 0.009 | flagellar hook protein |
| *lmo0701* | **-1.13** | 0.018 | **-1.33** | 0.043 | unknown protein |
| *lmo0702* | **-1.39** | 0.004 | **-2.45** | 0.005 | unknown protein |
| *flgK* | **-1.89** | 0.003 | **-1.62** | 0.022 | flagellar hook-associated protein |
| *fliD* | **-1.26** | 0.014 | **-1.55** | 0.055 | flagellar capping protein |
| *lmo0709* | **-2.09** | 0.002 | **-2.08** | 0.038 | unknown protein |
| *lmo0733* | **-1.63** | 0.008 | **-1.04** | 0.011 | similar to transcriptional regulators |
| *lmo0740* | **-1.51** | 0.015 | **-1.95** | 0.036 | similar to cyclic nucleotide-binding proteins (Crp-like) |
| *lmo0742* | **-1.08** | 0.026 | **-1.35** | 0.002 | similar to ABC transporter, ATP-binding protein |
| *lmo0754* | **-1.32** | 0.004 | **-1.10** | 0.016 | putative bile acid 7-alpha dehydratase |
| *lmo0781* | **-1.45** | 0.019 | **-1.21** | 0.002 | similar to PTS system, mannose-specific IID component |
| *lmo0782* | **-2.43** | 0.001 | **-1.06** | 0.015 | similar to PTS system, mannose-specific IIC component |
| *lmo0800* | **-4.04** | 0.001 | **-2.31** | 0.000 | similar to uncharacterized conserved proteins |
| *lmo0875* | **-2.13** | 0.000 | **-1.48** | 0.001 | similar to PTS system, cellobiose-specific IIB component |
| *lmo0911* | **-1.11** | 0.046 | **-1.19** | 0.044 | similar to uncharacterized conserved proteins |
| *lmo0917* | **-2.49** | 0.001 | **-1.17** | 0.001 | similar to beta-glucosidase |
| *lmo0994* | **-2.27** | 0.000 | **-2.14** | 0.000 | unknown protein |
| *lmo0996* | **-1.02** | 0.028 | **-1.08** | 0.004 | similar to methylated-DNA-protein-cystein methyltransferase |
| *clpE* | **-5.75** | 0.000 | **-3.36** | 0.001 | Clp protease ATP-binding subunit |
| *moaD* | **-2.43** | 0.039 | **-1.20** | 0.009 | similar to molybdopterin converting factor subunit 1 |
| *pdhA* | **-1.89** | 0.003 | **-2.60** | 0.000 | pyruvate dehydrogenase E1 component, alpha subunit |
| *pdhC* | **-1.35** | 0.045 | **-3.28** | 0.000 | pyruvate dehydrogenase E2 component (dihydrolipoamide acetyltransferase) |
| *cbiT* | **-1.08** | 0.035 | **-1.02** | 0.020 | precorrin-8W decarboxylase |
| *lmo1201* | **-1.21** | 0.039 | **-1.07** | 0.001 | uroporphyrin-III C-methyltransferase |
| *lmo1254* | **-1.27** | 0.010 | **-1.63** | 0.005 | similar to alpha,alpha-phosphotrehalase |
| *lmo1263* | **-1.09** | 0.007 | **-1.28** | 0.000 | putative transcriptional regulator |
| *lmo1348* | **-1.69** | 0.013 | **-2.06** | 0.001 | aminomethyltransferase |
| *lmo1349* | **-2.65** | 0.001 | **-2.88** | 0.000 | glycine dehydrogenase subunit 1 |
| *acoB* | **-1.11** | 0.011 | **-1.05** | 0.019 | 2-oxoisovalerate dehydrogenase beta subunit |
| *pflC* | **-6.12** | 0.000 | **-2.50** | 0.000 | pyruvate-formate lyase-activating enzyme |
| *lmo1534* | **-1.13** | 0.016 | **-1.52** | 0.004 | similar to L-lactate dehydrogenase |
| *lmo1580* | **-3.80** | 0.000 | **-2.67** | 0.000 | similar to universal stress protein UspA and related nucleotide-binding proteins |
| *lmo1716* | **-2.42** | 0.001 | **-1.76** | 0.010 | putative transcriptional regulator, TetR/AcrR family |
| *lmo1718* | **-4.21** | 0.000 | **-4.32** | 0.000 | similar to uncharacterized conserved proteins |
| *lmo1719* | **-4.12** | 0.000 | **-4.35** | 0.000 | similar to PTS system, lichenan/cellobiose-specific IIA component |
| *lmo1720* | **-2.87** | 0.001 | **-3.83** | 0.000 | similar to PTS system, lichenan/cellobiose-specific IIB component |
| *lmo1830* | **-1.29** | 0.006 | **-1.50** | 0.001 | similar to dehydrogenases with different specificities (related to short-chain alcohol dehydrogenases) |
| *alsD* | **-6.15** | 0.000 | **-2.88** | 0.000 | alpha-acetolactate decarboxylase |
| *lmo2066* | **-3.54** | 0.000 | **-1.62** | 0.003 | Unknown protein |
| *groES* | **-1.49** | 0.006 | **-1.20** | 0.001 | class I heat-shock protein (chaperonin) GroES |
| *atoAD* | **-1.60** | 0.005 | **-1.13** | 0.006 | similar to acyl CoA:acetate/3-ketoacid CoA transferase |
| *lmo2205* | **-3.17** | 0.000 | **-1.66** | 0.000 | phosphoglycerate mutase |
| *lmo2213* | **-1.23** | 0.009 | **-1.01** | 0.002 | similar to uncharacterized enzyme involved in biosynthesis of extracellular polysaccharides |
| *lmo2229* | **-2.92** | 0.000 | **-1.27** | 0.016 | similar to membrane carboxypeptidases (penicillin-binding proteins) |
| *lmo2257* | **-1.29** | 0.029 | **-1.27** | 0.001 | unknown protein |
| *gadC* | **-2.92** | 0.001 | **-4.77** | 0.001 | glutamate:gamma-aminobutyric acid antiporter |
| *gadB* | **-3.97** | 0.000 | **-4.26** | 0.000 | glutamate decarboxylase |
| *lmo2391* | **-1.78** | 0.002 | **-1.46** | 0.002 | predicted nucleoside-diphosphate-sugar epimerase |
| *lmo2453* | **-1.91** | 0.002 | **-1.22** | 0.001 | similar to predicted hydrolases |
| *lmo2454* | **-1.88** | 0.053 | **-1.89** | 0.008 | unknown protein |
| *lmo2599* | **-1.32** | 0.003 | **-1.44** | 0.000 | similar to cobalt/nickel ion ABC transporter, permease protein |
| *lmo2603* | **-2.45** | 0.003 | **-1.04** | 0.009 | predicted acetamidase |
| *adk* | **-1.75** | 0.002 | **-1.10** | 0.007 | adenylate kinase |
| *lmo2645* | **-2.99** | 0.000 | **-2.30** | 0.008 | unknown protein (unlisted in GenBank database) |
| *lmo2646* | **-4.18** | 0.000 | **-2.24** | 0.014 | similar to uncharacterized conserved proteins |
| *lmo2647* | **-3.91** | 0.000 | **-1.70** | 0.019 | creatinine amidohydrolase |
| *lmo2648* | **-4.93** | 0.000 | **-2.80** | 0.002 | similar to predicted metal-dependent hydrolases |
| *lmo2649* | **-4.76** | 0.000 | **-3.20** | 0.000 | similar to PTS system, mannitol/ascorbate-specific IIC component |
| *lmo2650* | **-3.92** | 0.000 | **-2.36** | 0.001 | similar to PTS system, mannitol/ascorbate-specific IIB component |
| *lmo2651* | **-2.18** | 0.020 | **-2.09** | 0.001 | similar to PTS system, mannitol/ascorbate-specific IIA component |
| *lmo2659* | **-2.33** | 0.000 | **-1.74** | 0.034 | ribulose-5-phosphate 3 epimerase |
| *lmo2660* | **-4.34** | 0.000 | **-1.98** | 0.046 | transketolase |
| *lmo2661* | **-3.90** | 0.000 | **-4.89** | 0.005 | similar to ribulose-5-phosphate 3 epimerase |
| *lmo2662* | **-4.58** | 0.011 | **-3.06** | 0.015 | ribose 5-phosphate isomerase B |
| *lmo2663* | **-5.28** | 0.009 | **-5.15** | 0.004 | polyol dehydrogenase |
| *lmo2664* | **-7.36** | 0.000 | **-5.26** | 0.002 | similar to sorbitol dehydrogenase |
| *lmo2665* | **-6.09** | 0.000 | **-5.08** | 0.001 | similar to PTS system, galacitol-specific IIC component |
| *lmo2666* | **-7.71** | 0.000 | **-6.39** | 0.000 | similar to PTS system, galacitol-specific IIB component |
| *lmo2667* | **-6.53** | 0.004 | **-4.60** | 0.000 | similar to PTS system, galacitol-specific IIA component |
| *lmo2668* | **-6.31** | 0.000 | **-5.86** | 0.000 | galacitol-specific PTS system operon regulator |
| *lmo2672* | **-2.22** | 0.000 | **-2.48** | 0.000 | similar to transcription regulator, AraC family |
| *lmo2673* | **-6.70** | 0.000 | **-1.79** | 0.007 | similar to universal stress protein UspA |
| *lmo2674* | **-5.79** | 0.000 | **-2.87** | 0.002 | ribose 5-phosphate isomerase B |
| *lmo2675* | **-1.09** | 0.010 | **-1.97** | 0.001 | unknown protein |
| *lmo2683* | **-10.15** | 0.000 | **-2.26** | 0.000 | similar to PTS system, cellobiose-specific IIB component |
| *lmo2684* | **-9.46** | 0.000 | **-4.14** | 0.000 | similar to PTS system, cellobiose-specific IIC component |
| *lmo2685* | **-8.68** | 0.000 | **-6.00** | 0.000 | similar to PTS system, cellobiose-specific IIA component |
| *lmo2687* | **-2.39** | 0.000 | **-3.50** | 0.000 | similar to FtsK/RodA/SpoIIIE and related proteins |
| *lmo2688* | **-1.97** | 0.002 | **-1.33** | 0.011 | similar to FtsK/RodA/SpoIIIE and related proteins |
| *lmo2693* | **-1.76** | 0.002 | **-1.21** | 0.001 | thymidylate kinase |
| *lmo2695* | **-4.42** | 0.000 | **-1.63** | 0.001 | similar to dihydroxyacetone kinase, N-terminal domain |
| *lmo2696* | **-3.49** | 0.000 | **-1.59** | 0.001 | similar to dihydroxyacetone kinase, C-terminal domain |
| *lmo2697* | **-2.01** | 0.002 | **-2.54** | 0.000 | putative PTS-dependent dihydroxyacetone kinase |
| *lmo2707* | **-10.26** | 0.000 | **-5.78** | 0.000 | unknown protein |
| *lmo2731* | **-4.33** | 0.003 | **-4.93** | 0.000 | unknown protein |
| *lmo2732* | **-5.03** | 0.000 | **-4.45** | 0.000 | similar to transcriptional regulator, RpiR-like |
| *lmo2742* | **-1.53** | 0.005 | **-1.36** | 0.000 | similar to uncharacterized conserved proteins |
| *lmo2755* | **-6.78** | 0.000 | **-5.29** | 0.000 | similar to predicted acyl esterases |
| *topB* | **-1.52** | 0.002 | **-1.17** | 0.006 | topoisomerase IA |
| *lmo2778* | **-6.20** | 0.000 | **-2.60** | 0.000 | unknown protein |
| *lmo2779* | **-5.58** | 0.000 | **-2.05** | 0.001 | similar to probable GTP-binding protein |
| *lmo2780* | **-5.77** | 0.000 | **-2.53** | 0.000 | similar to PTS system, cellobiose-specific IIA component |
| *lmo2781* | **-1.50** | 0.005 | **-1.58** | 0.000 | beta-glucosidase |
| *lmo2782* | **-5.03** | 0.000 | **-4.60** | 0.000 | similar to PTS system, cellobiose-specific IIB component |
| *lmo2801* | **-1.19** | 0.018 | **-1.02** | 0.008 | N-acetylmannosamine-6-phosphate epimerase |
| *lmo2803* | **-3.98** | 0.001 | **-2.64** | 0.001 | unknown protein |
| *lmo2805* | **-2.78** | 0.000 | **-2.62** | 0.000 | unknown protein |
| *lmo2812* | **-1.13** | 0.010 | **-1.25** | 0.001 | similar to D-alanyl-D-alanine carboxypeptidases |
| *lmo2817* | **-1.18** | 0.008 | **-1.65** | 0.004 | putative N-acyl-L-amino acid amidohydrolase/carboxypeptidase |
| *lmo2826* | **-1.73** | 0.001 | **-1.54** | 0.001 | predicted transporter protein |
| *lmo2827* | **-3.58** | 0.001 | **-2.23** | 0.003 | putative transcriptional regulator, MarR family |
| *lmo2828* | **-6.42** | 0.000 | **-4.71** | 0.000 | unknown protein |
| *lmo2830* | **-2.75** | 0.000 | **-1.22** | 0.002 | thioredoxin domain-containing protein |
| *lmo2832* | **-1.29** | 0.007 | **-1.92** | 0.004 | putative glycerate kinase |
| *lmo2851* | **-2.23** | 0.002 | **-1.58** | 0.000 | similar to transcription regulator, AraC family |
| *lmo2852* | **-4.27** | 0.001 | **-1.32** | 0.003 | similar to uncharacterized conserved proteins |
