# Supplementary material for: Characterisation of the Transcriptomes of Genetically Diverse Listeria monocytogenes Exposed to Hyperosmotic and Low Temperature Conditions Reveal Global Stress-Adaptation Mechanisms
Source: PLoS One. 2013 Sep 4;8(9):e73603. doi: 10.1371/journal.pone.0073603 (PMC3762727; doi:10.1371/journal.pone.0073603)
Supplement: Table S4 — Log ratios of significantly up-regulated genes in L. monocytogenes strain ScottA independently adapted to hyperosmotic stress induced by supplementing BHIB with 12% w/v salt or 4°C cold-temperature stress. * Gene nomenclature used as per L. monocytogenes EGD-e genome. Gene homologs and predicted functions were obtained collectively from variety of sources including circulating literature and web based databases. # LR: log ratio. Genes were considered significantly up-regulated with LR >1 which is equivocal of twofold up-regulation. ¥ Genes with P value >0.05 were not statistically significant and were excluded from this table. (DOCX) [file pone.0073603.s004.docx]

| Gene^*^ | Salt adapted | | Cold adapted | | Function |
| --- | --- | --- | --- | --- | --- |
|  | LR^#^ | P^¥^ | LR | P |  |
| *lmo0047* | **1.22** | 0.012 | **1.88** | 0.000 | predicted membrane protein |
| *metS* | **1.01** | 0.001 | **1.07** | 0.001 | methionyl-tRNA synthetase |
| *lmo0186* | **1.43** | 0.017 | **4.01** | 0.000 | similar to uncharacterized conserved proteins |
| *lmo0194* | **1.37** | 0.000 | **1.60** | 0.000 | similar to ABC transporter, ATP-binding protein |
| *lmo0195* | **1.20** | 0.001 | **2.07** | 0.000 | similar to putative ABC transporter, permease protein |
| *lmo0196* | **1.19** | 0.000 | **1.52** | 0.012 | similar to uncharacterized protein involved in the regulation of septum location |
| *gcaD* | **1.20** | 0.001 | **1.12** | 0.003 | glucosamine-1-phosphate N-acetyltransferase / UDP-N-acetylglucosamine pyrophosphorylase |
| *lmo0227* | **2.68** | 0.000 | **1.31** | 0.000 | putative tRNA-dihydrouridine |
| *rplK* | **1.49** | 0.000 | **2.54** | 0.006 | ribosomal protein L11 |
| *rplA* | **1.90** | 0.000 | **2.01** | 0.007 | ribosomal protein L1 |
| *rplJ* | **2.11** | 0.000 | **1.52** | 0.010 | ribosomal protein L10 |
| *rplL* | **1.96** | 0.000 | **1.36** | 0.002 | ribosomal protein L7/L12 |
| *lmo0485* | **1.07** | 0.004 | **1.27** | 0.003 | similar to nitroreductase-like family protein |
| *lmo1008* | **2.09** | 0.003 | **2.20** | 0.002 | unknown protein |
| *pycA* | **1.27** | 0.000 | **1.71** | 0.007 | pyruvate carboxylase |
| *guaA* | **1.04** | 0.003 | **1.42** | 0.003 | GMP synthase (glutamine hydrolyzing) |
| *lmo1183* | **1.31** | 0.003 | **1.47** | 0.000 | unknown protein |
| *lmo1245* | **1.30** | 0.000 | **2.15** | 0.000 | unknown protein |
| *lmo1255* | **1.49** | 0.001 | **2.04** | 0.000 | similar to PTS system, trehalose-specific IIBC component |
| *lmo1266* | **1.29** | 0.011 | **2.18** | 0.000 | unknown protein |
| *tig* | **1.10** | 0.009 | **1.85** | 0.012 | trigger factor (prolyl isomerase) |
| *topA* | **1.77** | 0.000 | **1.62** | 0.000 | topoisomerase IA |
| *lmo1306* | **3.21** | 0.001 | **3.53** | 0.001 | similar to uncharacterized conserved proteins |
| *lmo1308* | **1.08** | 0.001 | **1.68** | 0.000 | similar to predicted methyltransferases |
| *lmo1315* | **1.01** | 0.003 | **1.29** | 0.000 | undecaprenyl pyrophosphate synthase |
| *lmo1318* | **2.16** | 0.000 | **1.28** | 0.001 | putative membrane-associated Zn-dependent metalloprotease |
| *lmo1338* | **1.09** | 0.016 | **1.03** | 0.008 | unknown protein |
| *lmo1411* | **1.72** | 0.004 | **1.20** | 0.001 | similar to predicted transcriptional regulators |
| *lmo1431* | **2.02** | 0.000 | **2.10** | 0.000 | ABC transporter, ATP-binding protein |
| *lmo1528* | **1.03** | 0.009 | **1.62** | 0.001 | unknown protein |
| *yajC* | **1.69** | 0.003 | **3.09** | 0.005 | preprotein translocase YajC subunit |
| *lmo1530* | **1.19** | 0.001 | **1.10** | 0.006 | similar to queuine tRNA-ribosyltransferase |
| *thiI* | **1.49** | 0.002 | **1.48** | 0.002 | thiamine biosynthesis ATP pyrophosphatase |
| *rpsD* | **1.03** | 0.001 | **1.73** | 0.020 | ribosomal protein S4 |
| *lmo1707* | **1.10** | 0.000 | **1.81** | 0.000 | unknown protein |
| *lmo1722* | **1.09** | 0.002 | **1.32** | 0.000 | putative ATP-dependent RNA helicase |
| *rpmI* | **1.80** | 0.000 | **1.66** | 0.007 | ribosomal protein L35 |
| *infC* | **1.21** | 0.003 | **1.86** | 0.006 | translation initiation factor IF3 |
| *rplS* | **1.29** | 0.000 | **3.05** | 0.002 | ribosomal protein L19 |
| *acpA* | **1.36** | 0.020 | **3.18** | 0.013 | acyl carrier protein |
| *rpmB* | **3.01** | 0.001 | **3.58** | 0.007 | ribosomal protein L28 |
| *lmo1826* | **1.03** | 0.009 | **2.27** | 0.001 | RNA polymerase, omega subunit |
| *fur* | **1.42** | 0.000 | **2.16** | 0.000 | ferric uptake regulator |
| *lmo1979* | **1.28** | 0.001 | **1.12** | 0.000 | similar to uncharacterized conserved proteins |
| *lmo2029* | **1.05** | 0.021 | **1.11** | 0.002 | predicted integral membrane protein |
| *murG* | **1.57** | 0.000 | **1.20** | 0.002 | UDP-N-acetylglucosamine-N-acetylmuramyl-(pentapeptide) pyrophosphoryl-undecaprenol N-acetylglucosamine transferase |
| *murE* | **1.43** | 0.001 | **1.41** | 0.006 | UDP-N-acetylmuramoylalanyl-D-glutamate--2,6-diaminopimelate ligase |
| *lmo2042* | **1.14** | 0.008 | **1.36** | 0.000 | similar to uncharacterized conserved proteins |
| *lmo2048* | **2.59** | 0.000 | **1.75** | 0.001 | similar to uncharacterized conserved proteins |
| *lmo2050* | **2.15** | 0.000 | **1.05** | 0.007 | putative excinuclease ATPase subunit |
| *lmo2051* | **1.21** | 0.000 | **1.03** | 0.016 | similar to predicted secreted protein |
| *lmo2056* | **1.66** | 0.000 | **1.48** | 0.001 | similar to uncharacterized conserved proteins |
| *lmo2062* | **1.02** | 0.001 | **1.40** | 0.000 | similar to copper export proteins |
| *lmo2127* | **1.24** | 0.002 | **1.96** | 0.005 | CAAX amino terminal protease family protein |
| *lmo2186* | **1.06** | 0.016 | **1.98** | 0.000 | peptidoglycan bound protein; iron transport-associated domain |
| *oppE* | **2.26** | 0.000 | **1.15** | 0.026 | similar to oligopeptide ABC transporter, ATP binding protein |
| *oppC* | **1.46** | 0.000 | **1.31** | 0.034 | similar to oligopeptide ABC transporter, permease protein |
| *oppB* | **1.38** | 0.000 | **1.71** | 0.007 | similar to oligopeptide ABC transporter, permease protein |
| *oppA* | **1.54** | 0.002 | **1.62** | 0.020 | similar to oligopeptide ABC transporter, substrate binding protein |
| *prsA* | **2.16** | 0.001 | **3.19** | 0.005 | similar to post-translocation molecular chaperone |
| *lmo2223* | **2.74** | 0.004 | **1.89** | 0.029 | similar to uncharacterized conserved proteins |
| *lmo2241* | **1.53** | 0.008 | **1.68** | 0.001 | similar to transcription regulator, GntR family |
| *lmo2261* | **1.18** | 0.000 | **2.90** | 0.000 | similar to uncharacterized conserved proteins |
| *lmo2359* | **1.22** | 0.000 | **3.42** | 0.000 | similar to predicted hydrolases of the HAD superfamily |
| *lmo2369* | **1.27** | 0.003 | **1.75** | 0.000 | similar to B. subtilis general stress protein 13 containing a ribosomal S1 protein domain |
| *lmo2376* | **1.27** | 0.000 | **1.25** | 0.009 | similar to peptidyl-prolyl cis-trans isomerase |
| *lmo2428* | **1.08** | 0.001 | **2.12** | 0.000 | similar to FtsK/RodA/SpoIIIE and related proteins |
| *secG* | **1.25** | 0.001 | **2.43** | 0.000 | preprotein translocase SecG subunit |
| *lmo2479* | **1.14** | 0.001 | **1.27** | 0.000 | similar to uncharacterized conserved proteins |
| *spl* | **1.66** | 0.002 | **1.16** | 0.034 | putative cell wall-associated hydrolases (invasion-associated proteins); peptidoglycan lytic protein P45 |
| *ftsE* | **2.82** | 0.000 | **1.44** | 0.040 | cell division protein; ABC Transporter, permease protein |
| *lmo2522* | **3.90** | 0.000 | **4.40** | 0.000 | similar to uncharacterized conserved proteins |
| *hom* | **1.02** | 0.019 | **1.81** | 0.000 | homoserine dehydrogenase |
| *rpmE* | **1.95** | 0.000 | **3.32** | 0.004 | ribosomal protein L31 |
| *lmo2555* | **1.15** | 0.000 | **1.68** | 0.004 | similar to glycosyltransferases |
| *lmo2597* | **1.54** | 0.001 | **1.38** | 0.054 | ribosomal protein L13 |
| *lmo2843* | **1.36** | 0.001 | **2.15** | 0.000 | similar to uncharacterized protein involved in cytokinesis |
