# Supplementary material for: Characterisation of the Transcriptomes of Genetically Diverse Listeria monocytogenes Exposed to Hyperosmotic and Low Temperature Conditions Reveal Global Stress-Adaptation Mechanisms
Source: PLoS One. 2013 Sep 4;8(9):e73603. doi: 10.1371/journal.pone.0073603 (PMC3762727; doi:10.1371/journal.pone.0073603)
Supplement: Table S5 — Log ratios of significantly down-regulated genes in L. monocytogenes strain ScottA independently adapted to hyperosmotic stress induced by supplementing BHIB with 12% w/v salt or 4°C cold-temperature stress. * Gene nomenclature used as per L. monocytogenes EGD-e genome. Gene homologs and predicted functions were obtained collectively from variety of sources including circulating literature and web based databases. # LR: log ratio. Genes were considered significantly down-regulated with LR <-1 which is equivocal of twofold down-regulation. ¥ Genes with P value >0.05 were not statistically significant and were excluded from this table. (DOCX) [file pone.0073603.s005.docx]

| Gene^*^ | Salt adapted | | Cold adapted | | Function |
| --- | --- | --- | --- | --- | --- |
|  | LR^#^ | P^¥^ | LR | P |  |
| *lmo0029* | **-1.16** | 0.002 | **-1.30** | 0.000 | unknown protein |
| *lmo0043* | **-1.12** | 0.003 | **-1.77** | 0.003 | arginine deiminase |
| *agrB* | **-6.52** | 0.000 | **-1.87** | 0.000 | similar to S. aureus AgrB protein |
| *agrD* | **-5.51** | 0.000 | **-1.68** | 0.001 | putative autoinducing peptide |
| *mptC* | **-1.56** | 0.049 | **-2.25** | 0.013 | similar to PTS system, mannose-specific IIC component |
| *lmo0105* | **-2.52** | 0.009 | **-2.01** | 0.000 | similar to chitinase B |
| *lmo0110* | **-1.02** | 0.002 | **-1.92** | 0.001 | similar to esterase/lipase family proteins |
| *lmo0133* | **-2.14** | 0.001 | **-3.21** | 0.001 | similar to uncharacterized conserved proteins |
| *lmo0134* | **-2.01** | 0.004 | **-3.93** | 0.001 | similar to acetyltransferase (GNAT) family proteins |
| *lmo0230* | **-1.28** | 0.003 | **-2.19** | 0.000 | similar to uncharacterized conserved proteins |
| *lmo0231* | **-1.36** | 0.000 | **-2.15** | 0.000 | similar to arginine kinase |
| *inlH/inlC2* | **-2.39** | 0.000 | **-2.35** | 0.011 | internalin H |
| *lmo0265* | **-2.28** | 0.000 | **-3.49** | 0.003 | putative succinyl-diaminopimelate desuccinylase |
| *lmo0291* | **-1.55** | 0.000 | **-1.96** | 0.001 | similar to metal-dependent hydrolases of the beta-lactamase superfamily |
| *htrA* | **-1.83** | 0.000 | **-1.12** | 0.011 | similar to heat-shock protein htrA serine protease |
| *lmo0343* | **-2.47** | 0.002 | **-1.93** | 0.028 | similar to transaldolase |
| *lmo0344* | **-2.11** | 0.008 | **-2.29** | 0.023 | similar to dehydrogenases with different specificities |
| *lmo0345* | **-2.46** | 0.004 | **-2.94** | 0.022 | similar to ribose 5-phosphate isomerase B |
| *lmo0346* | **-2.75** | 0.002 | **-2.25** | 0.051 | similar to triosephosphate isomerase |
| *lmo0347* | **-1.87** | 0.016 | **-2.55** | 0.015 | similar to dihydroxyacetone kinase, C-terminal domain |
| *lmo0348* | **-2.30** | 0.005 | **-2.94** | 0.022 | similar to dihydroxyacetone kinase, N-terminal domain |
| *lmo0349* | **-1.54** | 0.056 | **-2.21** | 0.043 | Unknown protein |
| *lmo0406* | **-1.16** | 0.006 | **-1.00** | 0.006 | similar to lactoylglutathione lyase |
| *inlA* | **-2.66** | 0.000 | **-3.91** | 0.000 | internalin A |
| *lmo0515* | **-2.49** | 0.000 | **-2.44** | 0.013 | similar to universal stress protein UspA |
| *lmo0539* | **-1.38** | 0.004 | **-1.69** | 0.000 | tagatose 1,6-diphosphate aldolase |
| *lmo0574* | **-1.00** | 0.001 | **-1.19** | 0.007 | beta-glucosidase |
| *lmo0580* | **-1.09** | 0.002 | **-1.95** | 0.004 | similar to predicted esterases |
| *lmo0584* | **-1.58** | 0.001 | **-1.30** | 0.053 | similar to predicted permeases |
| *lmo0602* | **-1.94** | 0.000 | **-3.31** | 0.000 | similar to sortase and related acyltransferases |
| *lmo0606* | **-1.32** | 0.009 | **-1.30** | 0.015 | putative transcriptional regulator, MarR family |
| *lmo0610* | **-1.69** | 0.000 | **-1.45** | 0.003 | putative peptidoglycan bound protein |
| *lmo0628* | **-1.61** | 0.001 | **-3.53** | 0.005 | unknown protein |
| *lmo0629* | **-1.98** | 0.000 | **-2.28** | 0.009 | hydrolase, isochorismatase family |
| *lmo0654* | **-3.50** | 0.000 | **-2.20** | 0.005 | unknown protein |
| *flaA* | **-4.70** | 0.000 | **-5.08** | 0.000 | flagellin |
| *cheY* | **-1.55** | 0.000 | **-1.43** | 0.001 | chemotaxis response regulator |
| *fliY* | **-1.07** | 0.013 | **-1.49** | 0.002 | similar to flagellar switch protein |
| *flgD* | **-1.09** | 0.000 | **-2.18** | 0.000 | flagellar hook capping protein |
| *flgE* | **-1.12** | 0.001 | **-1.40** | 0.000 | flagellar hook protein |
| *fliN* | **-1.68** | 0.004 | **-1.62** | 0.028 | similar to flagellar motor switch protein |
| *lmo0702* | **-1.10** | 0.003 | **-1.61** | 0.001 | unknown protein |
| *lmo0704* | **-1.09** | 0.002 | **-1.49** | 0.004 | unknown protein |
| *fliS* | **-1.28** | 0.003 | **-1.62** | 0.009 | flagellin-specific chaperone |
| *flgC* | **-1.70** | 0.022 | **-1.20** | 0.037 | flagellar basal body rod protein |
| *fliE* | **-1.79** | 0.000 | **-2.01** | 0.000 | flagellar hook-basal body protein |
| *lmo0716* | **-1.06** | 0.012 | **-1.02** | 0.015 | flagellum-specific ATP synthase |
| *lmo0724* | **-1.39** | 0.003 | **-3.30** | 0.000 | similar to uncharacterized conserved proteins |
| *lmo0781* | **-2.03** | 0.000 | **-1.73** | 0.000 | similar to PTS system, mannose-specific IID component |
| *lmo0782* | **-2.63** | 0.000 | **-2.13** | 0.000 | similar to PTS system, mannose-specific IIC component |
| *lmo0794* | **-2.10** | 0.001 | **-2.91** | 0.004 | similar to putative NADH-flavin reductases |
| *lmo0796* | **-1.08** | 0.001 | **-3.26** | 0.000 | similar to uncharacterized conserved proteins |
| *lmo0800* | **-3.35** | 0.001 | **-1.57** | 0.016 | similar to uncharacterized conserved proteins |
| *lmo0911* | **-1.56** | 0.036 | **-1.85** | 0.012 | similar to uncharacterized conserved proteins |
| *lmo0953* | **-1.15** | 0.002 | **-1.45** | 0.000 | putative lipoprotein |
| *lmo0956* | **-1.23** | 0.002 | **-2.00** | 0.001 | N-acetylglucosamine-6-phosphate deacetylase |
| *lmo0983* | **-1.43** | 0.001 | **-1.44** | 0.000 | glutathione peroxidase |
| *lmo0996* | **-1.23** | 0.002 | **-1.29** | 0.002 | similar to methylated-DNA-protein-cystein methyltransferase |
| *clpE* | **-2.83** | 0.001 | **-3.83** | 0.000 | Clp protease ATP-binding subunit |
| *moaB* | **-1.07** | 0.009 | **-1.55** | 0.001 | similar to molybdenum cofactor biosynthesis protein B |
| *lmo1140* | **-1.07** | 0.014 | **-1.90** | 0.014 | similar to uncharacterized conserved proteins |
| *eutN* | **-1.99** | 0.047 | **-1.65** | 0.036 | similar to carboxysome shell protein |
| *pduO* | **-1.09** | 0.045 | **-1.32** | 0.000 | similar to ATP:cob(I)alamin adenosyltransferase |
| *pduF* | **-1.12** | 0.050 | **-1.89** | 0.006 | similar to glycerol uptake facilitator protein |
| *lmo1241* | **-1.17** | 0.007 | **-1.70** | 0.000 | similar to uncharacterized conserved proteins |
| *lmo1349* | **-1.36** | 0.001 | **-1.96** | 0.004 | glycine dehydrogenase subunit 1 |
| *rpoD* | **-1.41** | 0.018 | **-2.53** | 0.000 | RNA polymerase, primary sigma subunit (sigma70/sigma32) |
| *lmo1539* | **-1.26** | 0.022 | **-3.12** | 0.000 | similar to glycerol uptake facilitator |
| *lmo1580* | **-2.05** | 0.000 | **-1.46** | 0.014 | similar to universal stress protein UspA |
| *lmo1718* | **-1.54** | 0.005 | **-1.30** | 0.006 | similar to uncharacterized conserved proteins |
| *lmo1719* | **-1.72** | 0.000 | **-1.25** | 0.001 | similar to PTS system, lichenan/cellobiose-specific IIA component |
| *lmo1720* | **-1.58** | 0.000 | **-1.37** | 0.007 | similar to PTS system, lichenan/cellobiose-specific IIB component |
| *lmo1726* | **-1.35** | 0.018 | **-1.37** | 0.001 | similar to predicted dehydrogenases and related proteins |
| *trmD* | **-1.41** | 0.010 | **-1.35** | 0.040 | tRNA (Guanine-N1)-methyltransferase |
| *alsD* | **-3.53** | 0.000 | **-2.08** | 0.000 | alpha-acetolactate decarboxylase |
| *alsS* | **-1.86** | 0.012 | **-2.19** | 0.019 | alpha-acetolactate synthase |
| *lmo2096* | **-1.05** | 0.014 | **-1.15** | 0.001 | similar to PTS system, galacitol-specific IIC component |
| *lmo2098* | **-1.22** | 0.013 | **-1.35** | 0.002 | similar to PTS system, galacitol-specific IIA component |
| *lmo2205* | **-1.68** | 0.001 | **-3.13** | 0.000 | phosphoglycerate mutase |
| *lmo2213* | **-1.26** | 0.003 | **-2.36** | 0.000 | similar to uncharacterized enzyme involved in biosynthesis of extracellular polysaccharides |
| *lmo2253* | **-1.19** | 0.002 | **-1.31** | 0.005 | similar to phosphoglucomutases |
| *fruA* | **-3.93** | 0.000 | **-1.04** | 0.017 | similar to PTS system, fructose-specific IIABC component |
| *gadC* | **-4.88** | 0.000 | **-6.20** | 0.000 | glutamate:gamma-aminobutyric acid antiporter |
| *gadB* | **-5.11** | 0.000 | **-4.69** | 0.000 | glutamate decarboxylase |
| *lmo2391* | **-1.59** | 0.021 | **-2.77** | 0.002 | predicted nucleoside-diphosphate-sugar epimerase |
| *gadA* | **-1.46** | 0.010 | **-2.63** | 0.000 | glutamate decarboxylase |
| *lmo2454* | **-3.54** | 0.003 | **-2.62** | 0.055 | unknown protein |
| *lmo2571* | **-1.56** | 0.009 | **-1.82** | 0.001 | similar to nicotinamidase |
| *lmo2572* | **-1.61** | 0.011 | **-2.67** | 0.001 | reductase family protein |
| *lmo2573* | **-1.64** | 0.001 | **-2.40** | 0.000 | alcohol dehydrogenase, zinc-dependent |
| *lmo2664* | **-1.60** | 0.006 | **-1.23** | 0.001 | similar to sorbitol dehydrogenase |
| *lmo2666* | **-1.59** | 0.007 | **-1.45** | 0.002 | similar to PTS system, galacitol-specific IIB component |
| *lmo2667* | **-1.96** | 0.000 | **-1.09** | 0.003 | similar to PTS system, galacitol-specific IIA component |
| *lmo2668* | **-1.75** | 0.005 | **-1.23** | 0.000 | galacitol-specific PTS system operon regulator |
| *lmo2673* | **-3.80** | 0.000 | **-2.65** | 0.027 | similar to universal stress protein UspA |
| *lmo2674* | **-3.73** | 0.000 | **-2.13** | 0.011 | ribose 5-phosphate isomerase B |
| *lmo2684* | **-3.09** | 0.001 | **-1.11** | 0.007 | similar to PTS system, cellobiose-specific IIC component |
| *lmo2685* | **-2.01** | 0.000 | **-1.42** | 0.003 | similar to PTS system, cellobiose-specific IIA component |
| *lmo2695* | **-2.38** | 0.001 | **-3.49** | 0.000 | similar to dihydroxyacetone kinase, N-terminal domain |
| *lmo2696* | **-2.12** | 0.000 | **-2.87** | 0.003 | similar to dihydroxyacetone kinase, C-terminal domain |
| *lmo2697* | **-1.75** | 0.001 | **-3.48** | 0.000 | putative PTS-dependent dihydroxyacetone kinase |
| *lmo2708* | **-1.93** | 0.001 | **-1.36** | 0.030 | similar to PTS system, cellobiose-specific IIC component |
| *lmo2719* | **-1.81** | 0.001 | **-2.58** | 0.000 | similar to cytosine/adenosine deaminase protein family |
| *lmo2720* | **-1.26** | 0.005 | **-2.47** | 0.000 | acetyl-CoA synthetase |
| *lmo2742* | **-1.11** | 0.017 | **-1.54** | 0.000 | similar to uncharacterized conserved proteins |
| *lmo2792* | **-1.55** | 0.012 | **-1.14** | 0.004 | putative transcriptional regulator |
