# Supplementary material for: Characterisation of the Transcriptomes of Genetically Diverse Listeria monocytogenes Exposed to Hyperosmotic and Low Temperature Conditions Reveal Global Stress-Adaptation Mechanisms
Source: PLoS One. 2013 Sep 4;8(9):e73603. doi: 10.1371/journal.pone.0073603 (PMC3762727; doi:10.1371/journal.pone.0073603)
Supplement: Table S6 — Log ratios of significantly up-regulated genes in L. monocytogenes strain 70-1700 independently adapted to hyperosmotic stress induced by supplementing BHIB with 8% w/v salt or 4°C cold-temperature stress. * Gene nomenclature used as per L. monocytogenes EGD-e genome. Gene homologs and predicted functions were obtained collectively from variety of sources including circulating literature and web based databases. # LR: log ratio. Genes were considered significantly up-regulated with LR >1 which is equivocal of twofold up-regulation. ¥ Genes with P value >0.05 were not statistically significant and were excluded from this table. (DOCX) [file pone.0073603.s006.docx]

| Gene^*^ | Salt adapted | | Cold adapted | | Function |
| --- | --- | --- | --- | --- | --- |
|  | LR^#^ | P^¥^ | LR | P |  |
| *ssb* | **1.74** | 0.003 | **1.52** | 0.000 | single-stranded DNA-binding protein |
| *spoVG* | **1.06** | 0.001 | **1.27** | 0.000 | uncharacterized protein involved in regulation of septum location |
| *gcaD* | **1.34** | 0.000 | **1.15** | 0.000 | glucosamine-1-phosphate N-acetyltransferase |
| *prs* | **1.39** | 0.000 | **1.15** | 0.000 | phosphoribosyl pyrophosphate synthetase |
| *lmo0227* | **1.32** | 0.003 | **1.64** | 0.000 | putative tRNA-dihydrouridine |
| *rplK* | **1.79** | 0.000 | **3.16** | 0.000 | ribosomal protein L11 |
| *rplA* | **1.08** | 0.002 | **2.97** | 0.000 | ribosomal protein L1 |
| *rplJ* | **1.52** | 0.000 | **3.03** | 0.000 | ribosomal protein L10 |
| *rplL* | **1.21** | 0.001 | **2.43** | 0.000 | ribosomal protein L7/L12 |
| *tig* | **1.59** | 0.000 | **1.01** | 0.014 | trigger factor (prolyl isomerase) |
| *lmo1271* | **1.04** | 0.001 | **1.68** | 0.000 | signal peptidase I |
| *luxS* | **1.12** | 0.003 | **1.84** | 0.000 | autoinducer-2 production protein luxS |
| *proS* | **1.26** | 0.000 | **1.12** | 0.001 | prolyl-tRNA synthetase |
| *pnpA* | **1.11** | 0.012 | **1.53** | 0.000 | polyribonucleotide nucleotidyltransferase |
| *nusB* | **1.08** | 0.005 | **1.09** | 0.002 | transcription termination factor |
| *lmo1431* | **1.37** | 0.000 | **1.97** | 0.000 | ABC transporter, ATP-binding protein |
| *lmo1440* | **1.16** | 0.011 | **1.18** | 0.001 | similar to uncharacterized conserved proteins |
| *lmo1452* | **1.15** | 0.004 | **1.35** | 0.000 | similar to uncharacterized conserved proteins |
| *lmo1463* | **1.06** | 0.001 | **1.67** | 0.000 | cytidine deaminase |
| *rpsT* | **1.66** | 0.024 | **2.17** | 0.000 | ribosomal protein S20 |
| *lmo1530* | **1.01** | 0.005 | **1.20** | 0.000 | similar to queuine tRNA-ribosyltransferase |
| *lmo1541* | **1.08** | 0.001 | **2.42** | 0.000 | predicted ribosomal protein |
| *lmo1582* | **1.27** | 0.000 | **2.40** | 0.000 | putative adenine-specific DNA methylase |
| *rpsD* | **1.48** | 0.000 | **1.99** | 0.000 | ribosomal protein S4 |
| *lmo1644* | **1.31** | 0.001 | **1.92** | 0.000 | putative DNA/RNA helicase, Snf2 family |
| *tsf* | **1.03** | 0.000 | **1.45** | 0.000 | elongation factor EF-Ts |
| *lmo1707* | **1.08** | 0.000 | **1.26** | 0.001 | unknown protein |
| *lmo1743* | **1.10** | 0.001 | **1.37** | 0.001 | unknown protein |
| *lmo1760* | **1.46** | 0.002 | **1.81** | 0.000 | predicted phosphate-binding enzyme |
| *rpmI* | **1.38** | 0.000 | **2.32** | 0.000 | ribosomal protein L35 |
| *rpmB* | **1.84** | 0.000 | **4.11** | 0.000 | ribosomal protein L28 |
| *lmo1818* | **1.03** | 0.011 | **1.54** | 0.000 | ribulose-5-phosphate 3 epimerase |
| *lmo1826* | **1.11** | 0.003 | **2.09** | 0.000 | RNA polymerase, omega subunit |
| *cca* | **1.48** | 0.001 | **1.15** | 0.000 | tRNA nucleotidyltransferase (CCA-adding enzyme) |
| *lmo1921* | **1.58** | 0.000 | **2.66** | 0.000 | unknown protein |
| *lmo1922* | **1.94** | 0.000 | **1.59** | 0.000 | similar to pilus assembly protein |
| *aroE* | **1.92** | 0.000 | **2.27** | 0.000 | 5-enolpyruvylshikimate-3-phosphate synthase |
| *resD* | **2.13** | 0.008 | **1.57** | 0.000 | two-component response regulator |
| *fur* | **1.83** | 0.010 | **2.13** | 0.000 | ferric uptake regulator |
| *glmS* | **1.39** | 0.000 | **1.05** | 0.000 | glucosamine--fructose-6-phosphate aminotransferase (isomerizing), putative |
| *lmo2048* | **1.64** | 0.000 | **2.09** | 0.000 | similar to uncharacterized conserved proteins |
| *lmo2056* | **1.44** | 0.001 | **1.51** | 0.000 | similar to uncharacterized conserved proteins |
| *lmo2071* | **1.20** | 0.000 | **1.01** | 0.002 | Unknown protein |
| *argG* | **2.26** | 0.000 | **1.01** | 0.001 | argininosuccinate synthase |
| *argH* | **1.52** | 0.001 | **1.48** | 0.000 | argininosuccinate lyase |
| *lmo2095* | **1.56** | 0.000 | **1.93** | 0.000 | similar to 1-phosphofructokinase |
| *pta* | **1.70** | 0.006 | **1.66** | 0.000 | phosphate acetyltransferase |
| *lmo2104* | **1.36** | 0.000 | **1.25** | 0.001 | similar to Fe2+ transport system protein A |
| *lmo2110* | **1.45** | 0.000 | **2.09** | 0.000 | mannose-6-phosphate isomerase |
| *lmo2111* | **1.22** | 0.001 | **1.52** | 0.000 | similar to NADPH-dependent flavin reductases and oxygen-insensitive nitroreductases |
| *oppE* | **1.63** | 0.000 | **1.85** | 0.000 | similar to oligopeptide ABC transporter, ATP binding protein |
| *oppC* | **1.43** | 0.000 | **1.74** | 0.000 | similar to oligopeptide ABC transporter, permease protein |
| *oppB* | **1.17** | 0.000 | **1.72** | 0.000 | similar to oligopeptide ABC transporter, permease protein |
| *lmo2199* | **1.31** | 0.005 | **1.17** | 0.001 | similar to predicted redox protein, regulator of disulfide bond formation |
| *lmo2207* | **1.66** | 0.000 | **1.56** | 0.000 | similar to putative integral membrane protein that interacts with FtsH |
| *lmo2223* | **1.37** | 0.000 | **1.10** | 0.001 | similar to uncharacterized conserved proteins |
| *lmo2227* | **1.06** | 0.002 | **1.36** | 0.004 | similar to ABC transporter, ATP-binding protein |
| *fruB* | **2.36** | 0.000 | **1.14** | 0.001 | fructose-1-phosphate kinase |
| *lmo2376* | **1.39** | 0.000 | **1.20** | 0.000 | similar to peptidyl-prolyl cis-trans isomerase |
| *lmo2428* | **1.24** | 0.000 | **2.47** | 0.000 | similar to FtsK/RodA/SpoIIIE and related proteins |
| *secG* | **1.42** | 0.037 | **1.59** | 0.000 | preprotein translocase SecG subunit |
| *lmo2471* | **1.08** | 0.002 | **1.09** | 0.000 | similar to NADH oxidase |
| *lmo2479* | **1.47** | 0.001 | **1.31** | 0.001 | similar to uncharacterized conserved proteins |
| *lgt* | **1.38** | 0.000 | **1.01** | 0.000 | prolipoprotein diacylglyceryl transferase |
| *lmo2491* | **1.19** | 0.003 | **1.06** | 0.000 | similar to predicted hydrolases of HD superfamily |
| *ftsX* | **1.49** | 0.007 | **1.24** | 0.003 | cell division protein; ABC Transporter, ATP-binding protein |
| *lmo2522* | **3.52** | 0.000 | **4.30** | 0.000 | similar to uncharacterized conserved proteins |
| *lmo2540* | **1.20** | 0.022 | **1.83** | 0.003 | putative protein-tyrosine-phosphatase |
| *rpmE* | **1.91** | 0.000 | **3.69** | 0.000 | ribosomal protein L31 |
| *lmo2555* | **1.13** | 0.000 | **1.09** | 0.000 | similar to glycosyltransferases |
| *rpsI* | **1.03** | 0.001 | **2.14** | 0.000 | ribosomal protein S9 |
| *rpmD* | **1.51** | 0.005 | **2.15** | 0.000 | ribosomal protein L30 |
| *rplR* | **1.37** | 0.000 | **2.21** | 0.000 | ribosomal protein L18 |
| *rpsH* | **1.69** | 0.000 | **2.38** | 0.000 | ribosomal protein S8 |
| *rplE* | **1.40** | 0.000 | **2.29** | 0.000 | ribosomal protein L5 |
| *rplN* | **1.54** | 0.000 | **2.23** | 0.000 | ribosomal protein L14 |
| *rpmC* | **1.24** | 0.008 | **1.88** | 0.000 | ribosomal protein L29 |
| *rpsS* | **1.18** | 0.000 | **2.40** | 0.000 | ribosomal protein S19 |
| *rplC* | **1.06** | 0.015 | **1.55** | 0.000 | ribosomal protein L3 |
| *fus* | **1.10** | 0.002 | **2.44** | 0.000 | elongation factor EF-G |
| *rpsL* | **1.43** | 0.000 | **3.24** | 0.000 | ribosomal protein S12 |
| *lmo2692* | **1.17** | 0.004 | **1.80** | 0.000 | unknown protein |
