# Supplementary material for: Characterisation of the Transcriptomes of Genetically Diverse Listeria monocytogenes Exposed to Hyperosmotic and Low Temperature Conditions Reveal Global Stress-Adaptation Mechanisms
Source: PLoS One. 2013 Sep 4;8(9):e73603. doi: 10.1371/journal.pone.0073603 (PMC3762727; doi:10.1371/journal.pone.0073603)
Supplement: Table S7 — Log ratios of significantly down-regulated genes in L. monocytogenes strain 70-1700 independently adapted to hyperosmotic stress induced by supplementing BHIB with 8% w/v salt or 4°C cold-temperature stress. * Gene nomenclature used as per L. monocytogenes EGD-e genome. Gene homologs and predicted functions were obtained collectively from variety of sources including circulating literature and web based databases. # LR: log ratio. Genes were considered significantly down-regulated with LR <-1 which is equivocal of twofold down-regulation. ¥ Genes with P value >0.05 were not statistically significant and were excluded from this table. (DOCX) [file pone.0073603.s007.docx]

| Gene* | Salt adapted | | Cold adapted | | Function |
| --- | --- | --- | --- | --- | --- |
|  | LR^#^ | P^¥^ | LR | P |  |
| *lmo0008* | **-1.21** | 0.008 | **-2.20** | 0.000 | similar to cardiolipin synthase |
| *lmo0030* | **-1.11** | 0.001 | **-1.04** | 0.000 | predicted hydrolase of the HAD superfamily |
| *lmo0032* | **-1.54** | 0.000 | **-1.70** | 0.000 | putative transcriptional regulator/sugar kinase |
| *lmo0057* | **-1.52** | 0.000 | **-1.67** | 0.000 | predicted membrane protein |
| *lmo0059* | **-1.08** | 0.004 | **-1.61** | 0.000 | unknown protein |
| *lmo0076* | **-1.17** | 0.004 | **-1.15** | 0.000 | methylated-DNA-[protein]-cysteine S-methyltransferase |
| *lmo0085* | **-1.12** | 0.019 | **-1.08** | 0.000 | similar to cyclic nucleotide-binding proteins (Crp-like) |
| *lmo0086* | **-1.45** | 0.001 | **-1.21** | 0.000 | unknown protein |
| *atpD* | **-1.10** | 0.001 | **-1.14** | 0.000 | F0F1-type ATP synthase, beta subunit |
| *lmo0127* | **-1.10** | 0.003 | **-1.42** | 0.021 | similar to protein gp20 from Bacteriophage A118 |
| *lmo0241* | **-1.08** | 0.018 | **-1.01** | 0.006 | putative tRNA/rRNA methylmethylase |
| *lmo0265* | **-1.91** | 0.000 | **-3.34** | 0.000 | putative succinyl-diaminopimelate desuccinylase |
| *lmo0274* | **-1.16** | 0.010 | **-1.29** | 0.005 | similar to cyclic nucleotide-binding proteins (Crp-like) |
| *lmo0291* | **-1.33** | 0.000 | **-2.26** | 0.000 | similar to metal-dependent hydrolases of the beta-lactamase superfamily |
| *htrA* | **-1.32** | 0.000 | **-1.55** | 0.001 | similar to heat-shock protein htrA serine protease |
| *lmo0308* | **-1.51** | 0.000 | **-1.22** | 0.024 | unknown protein |
| *lmo0318* | **-1.29** | 0.000 | **-1.05** | 0.047 | thiamine-phosphate pyrophosphorylase |
| *lmo0325* | **-1.06** | 0.000 | **-1.20** | 0.000 | similar to helix-turn-helix XRE-family like proteins |
| *lmo0331* | **-1.46** | 0.000 | **-1.01** | 0.002 | putative peptidoglycan bound protein |
| *lmo0353* | **-1.09** | 0.000 | **-1.13** | 0.000 | similar to acetyltransferases |
| *iolR* | **-1.25** | 0.001 | **-1.36** | 0.011 | similar to B. subtilis transcription repressor of myo-inositol catabolism operon |
| *lmo0388* | **-1.61** | 0.000 | **-1.19** | 0.004 | unknown protein |
| *lmo0402* | **-1.27** | 0.001 | **-2.72** | 0.000 | fructose-specific PTS system operon regulator |
| *lmo0409* | **-1.30** | 0.000 | **-1.27** | 0.000 | putative peptidoglycan bound protein (LPXTG motif) |
| *lmo0410* | **-1.56** | 0.000 | **-1.04** | 0.001 | similar to phosphoenolpyruvate synthase, C-terminal part |
| *lmo0417* | **-1.65** | 0.000 | **-1.29** | 0.001 | unknown protein |
| *lmo0425* | **-1.43** | 0.000 | **-1.89** | 0.000 | mannose/fructose-specific PTS system operon regulator |
| *lmo0450* | **-1.37** | 0.000 | **-1.75** | 0.000 | similar to uncharacterized conserved proteins |
| *lmo0496* | **-1.18** | 0.000 | **-1.54** | 0.000 | similar to uncharacterized conserved proteins |
| *lmo0537* | **-1.78** | 0.000 | **-1.27** | 0.003 | similar to N-carbamyl-L-amino acid amidohydrolase |
| *lmo0601* | **-1.56** | 0.000 | **-1.29** | 0.000 | similar to uncharacterized conserved proteins |
| *lmo0613* | **-1.26** | 0.001 | **-1.39** | 0.000 | alcohol dehydrogenase, zinc-dependent |
| *lmo0617* | **-1.24** | 0.001 | **-1.58** | 0.000 | putative lipoprotein |
| *lmo0709* | **-1.41** | 0.000 | **-1.38** | 0.000 | unknown protein |
| *lmo0721* | **-1.05** | 0.001 | **-1.23** | 0.000 | fibronectin-binding protein |
| *lmo0733* | **-1.31** | 0.000 | **-1.17** | 0.021 | similar to transcriptional regulators |
| *lmo0771* | **-1.32** | 0.000 | **-2.17** | 0.000 | unknown protein |
| *lmo0842* | **-1.39** | 0.002 | **-1.41** | 0.000 | putative peptidoglycan bound protein (LPXTG motif) |
| *lmo0875* | **-1.06** | 0.002 | **-2.03** | 0.000 | similar to PTS system, cellobiose-specific IIB component |
| *lmo0905* | **-1.51** | 0.000 | **-1.16** | 0.000 | similar to predicted protein tyrosine phosphatase |
| *lmo0930* | **-1.35** | 0.000 | **-1.20** | 0.000 | similar to metal-dependent hydrolases of the beta-lactamase superfamily III |
| *lmo0953* | **-2.61** | 0.000 | **-4.99** | 0.000 | putative lipoprotein |
| *lmo0993* | **-1.17** | 0.002 | **-1.38** | 0.000 | similar to Trk-type K+ transport systems, membrane component |
| *glpD* | **-1.17** | 0.003 | **-2.20** | 0.000 | glycerol-3-phosphate dehydrogenase |
| *aceF* | **-1.08** | 0.002 | **-1.21** | 0.001 | similar to branched-chain alpha-keto acid dehydrogenase E2 subunit (lipoamide acyltransferase) |
| *pflB* | **-1.65** | 0.000 | **-1.70** | 0.000 | pyruvate-formate lyase |
| *mreD* | **-1.61** | 0.000 | **-1.10** | 0.020 | similar to cell-shape determining protein |
| *radC* | **-1.01** | 0.001 | **-1.21** | 0.000 | similar to DNA repair protein |
| *hemL* | **-1.03** | 0.002 | **-1.11** | 0.003 | glutamate-1-semialdehyde 2,1-aminomutase |
| *lmo1865* | **-1.02** | 0.001 | **-1.08** | 0.000 | putative signal-transduction protein with CBS domains |
| *lmo2269* | **-1.51** | 0.002 | **-1.57** | 0.000 | unknown protein |
| *gadB* | **-1.05** | 0.015 | **-2.63** | 0.003 | glutamate decarboxylase |
| *lmo2684* | **-1.58** | 0.007 | **-2.85** | 0.000 | similar to PTS system, cellobiose-specific IIC component |
| *lmo2685* | **-1.23** | 0.000 | **-2.86** | 0.000 | similar to PTS system, cellobiose-specific IIA component |
| *lmo2792* | **-1.65** | 0.001 | **-2.14** | 0.000 | putative transcriptional regulator |
| *lmo2813* | **-1.15** | 0.002 | **-1.12** | 0.000 | unknown protein |
